# Genome-wide characterization and expression analysis of the growth-regulating factor family in *Saccharum*

**DOI:** 10.1186/s12870-022-03891-4

**Published:** 2022-11-02

**Authors:** Zilin Wu, Xinglong Chen, Danwen Fu, Qiaoying Zeng, Xiaoning Gao, Nannan Zhang, Jiayun Wu

**Affiliations:** 1 Guangdong Sugarcane Genetic Improvement Engineering Centre, Institute of Nanfan & Seed Industry, Guangdong Academy of Sciences, 510316 Guangzhou, Guangdong China; 2 Zhanjiang Research Center, Institute of Nanfan & Seed Industry, Guangdong Academy of Sciences, 524300 Zhanjiang, Guangdong China

**Keywords:** Growth-regulating factors, Gene expression, Abiotic stress, Growing development

## Abstract

**Background:**

Growth regulating factors (GRFs) are transcription factors that regulate diverse biological and physiological processes in plants, including growth, development, and abiotic stress. Although *GRF* family genes have been studied in a variety of plant species, knowledge about the identification and expression patterns of *GRFs* in sugarcane (*Saccharum* spp.) is still lacking.

**Results:**

In the present study, a comprehensive analysis was conducted in the genome of wild sugarcane (*Saccharum spontaneum*) and 10 *SsGRF* genes were identified and characterized. The phylogenetic relationship, gene structure, and expression profiling of these genes were analyzed entirely under both regular growth and low-nitrogen stress conditions. Phylogenetic analysis suggested that the 10 *SsGRF* members were categorized into six clusters. Gene structure analysis indicated that the *SsGRF* members in the same group were greatly conserved. Expression profiling demonstrated that most *SsGRF* genes were extremely expressed in immature tissues, implying their critical roles in sugarcane growth and development. Expression analysis based on transcriptome data and real-time quantitative PCR verification revealed that *GRF1* and *GRF3* were distinctly differentially expressed in response to low-nitrogen stress, which meant that they were additional participated in sugarcane stress tolerance.

**Conclusion:**

Our study provides a scientific basis for the potential functional prediction of *SsGRF* and will be further scrutinized by examining their regulatory network in sugarcane development and abiotic stress response, and ultimately facilitating their application in cultivated sugarcane breeding.

**Supplementary Information:**

The online version contains supplementary material available at 10.1186/s12870-022-03891-4.

## Background

Growth-regulating factors (GRFs) are plant-specific transcription factors, which play an essential role in regulating plant growth and abiotic stress response [1–3]. There are two conserved domains involved in GRF proteins: QLQ (Gln, Leu, and Gln) and WRC (Trp, Arg, and Cys), and both of them are located in the N-terminal region [4, 5]. The QLQ domain and the GRF-interacting factor (GIF) act upon each other, and it is also known as an important protein-protein interaction domain [6, 7]. The WRC domain contains a zinc finger motif and a functional nuclear localization signal that plays a role in DNA binding [6]. The C-terminal of GRF is variable and contains several low conservative motifs [5, 8]. *OsGRF1* is the first member of the identified *GRF* family, which is isolated from rice and plays a crucial role in gibberellin (GA)-induced stem elongation [4]. The *GRF* genes have recently been identified and characterized at genome-wide levels in many plant species, including *Arabidopsis* [9], rice [6], maize [5], tomato [8], soybean [2], cotton [10], and tea [11].

The *GRF* gene family plays a crucial role in plant growth and development, including root growth [12], stem elongation [13], leaf size and longevity [14–16], and flowering [17]. For instance, overexpression of *AtGRF1*, *AtGRF2*, and *AtGRF5* in *Arabidopsis* accounts for cotyledon and leaf size, and *AtGRF8* participates in flower development [14, 18], whereas *AtGRF9* has observably delayed leaf growth [15]. The overexpression of *BnGRF2* in rapeseed (*Brassica napus*) increases leave size, seed weight, and oil content [19]. The overexpression of *OsGRF1* in rice causes leaf distortion, lagged flowering, and incomplete development of carpels [4], *OsGRF4* plays a positive role in grain length, grain width, and grain weight [20], and *OsGRF6* plays an up-regulation role in auxin synthesis, increasing spike number, and promoting inflorescence development [21]. Furthermore, *GRF* genes are involved in plant response to abiotic stress [16, 22, 23]. For instance, the *AtGRF7* gene participates in increasing tolerance to salt and drought stress in *Arabidopsis* [1]. The *AtGRF1* and *AtGRF3* are implicated in the defensive reaction and disease tolerant processes [22, 24]. Expression patterns of *GhGRF1*and *GhGRF17* in cotton are changed under salt stress [10]. The whole *GmGRF* genes in soybean are markedly negatively regulated under shade stress [2]. Even though several studies have demonstrated that the *GRF* genes play an imperative role in regulating plant growth and abiotic stress, the information on *Saccharum* is still scarce.

Cultivated sugarcane (*Saccharum* spp.) is an important sugar and energy crop that is cultivated in tropical and subtropical regions of the world with high economic value [25]. Commercial sugarcane cultivars are interspecific hybrids derived from the hybridization of *S. spontaneum* (2n = 5x-16x = 40-128; x = 8) and *S. officinarum* (2n = 8x = 80, x = 10), followed by multigenerational backcrossing of the descendant with *S. officinarum* [26, 27]. However, during cultivation, it is vulnerable to extreme weather conditions or unfavorable environmental stress. Therefore, it is of great significance to study the molecular mechanism of sugarcane stress resistance and adaptation. Stress is a vital environmental factor that inhibits plant growth, yield, and quality. Over time, plants have gradually evolved mechanisms to withstand stress. As much as 90% of sugarcane dry matter is nitrogen [28], and nitrogen use efficiency (NUE) is quite low. During harvest, only a fifth (or less) of the nitrogen input comes from sugarcane dry biomass [29]. To improve NUE through conventional breeding and genetic engineering, it is essential to understand how nitrogen is used. The published *S. spontaneum* genome provides the ability to perform identification of the genome-wide genetic basis for desirable agronomic traits and stress-resistance genes in sugarcane [30].

In the present study, genome-wide identification and characterization of *GRF* family members in wild sugarcane were comprehensively conducted based on *S. spontaneum* genome data. Subsequently, the expression patterns of *GRFs* in different tissues, and under low-nitrogen stress were analyzed based on RNA-seq data. The results provide a set of informatics analyses of *GRFs* in the *Saccharum* and new insight into exploring and predicting the function of GRF proteins concerning the regulation of plant growth and abiotic stress to improve sugarcane agronomic traits through genetic modification.

## Materials and methods

### Genome-wide identification of ***GRF*** genes in ***S. spontaneum***

Genome-wide data for wild sugarcane (*S. spontaneum* cv AP85-441) were downloaded from the Ming laboratory database [30]. The amino acid sequences of 9, 12, 10, and 15 *GRF* genes in *Arabidopsis thaliana*, *Sorghum bicolor*, *Zea mays*, and *Oryza sativa* were downloaded from Phytozome v13 (https://phytozome-next.jgi.doe.gov/) and used as query sequences for a BlastP search of the *S. spontaneum* genome database. To verify the identified genes, the Hidden Markov Model (HMM) profiles of two GRF conserved domains WRC (PF08879) and QLQ (PF08880) were downloaded from the Pfam database (http://pfam.xfam.org/) [31], and then all non-redundant protein sequences were checked with the E-value lower than 1 × 10^− 10^ using HMMER v3.0 software (v3.2) [32]. The redundant sequences were deleted to retain the longest protein sequence. The *GRF* genes were named following the nomenclature scheme proposed by Schilling [33]. The NCBI-CDD database (https://www.ncbi.nlm.nih.gov/Structure/bwrpsb/bwrpsb.cgi) and the SMART database (http://smart.embl-heidelberg.de) were further used to analyze the domains of the candidate GRF proteins [34].

### Phylogenetic and gene duplication analysis

The protein sequences of GRFs from *A. thaliana*, *S. bicolor*, *Z. mays*, *O. sativa*, and *S. spontaneum* were conducted in multiple comparisons based on ClustalW software [35]. The subsequent phylogenetic tree was created with the neighbor-joining (NJ) algorithm with bootstrap analysis for 1,000 repetitions using MEGA X software [36]. The iTOL online tool (https://itol.embl.de/itol.cgi) [37] was used for visualizing and editing the phylogenetic trees. The MCScanX software [38] was used to determine *GRF* gene duplication events in interspecies and intraspecies. Enrichment analysis was used to depict the homology relationships between the number of gene families and a particular genome-wide duplication mode with Fisher’s exact test [38]. The synonymous substitution rate (Ka) and nonsynonymous substitution rate (Ks) values of duplicated gene pairs were calculated using KaKs_calculator 2.0 [39]. The selection pressure was determined by comparing the Ka/Ks ratio of orthologous *GRF* pairs between sugarcane and sorghum. The Ks value was translated into divergence time (T) in millions of years based on the rate of λ substitutions per synonymous site per year. The duplication time was calculated as follows: T = Ks/ (2 × λ) ×10^− 6^ Mya (λ = 6.1 × 10^–9^) [40]. Visualization of synteny diagrams and gene locations of *GRF* genes was performed using Circos software v0.69 [41].

### Characterization of GRF protein and gene structure

The isoelectric point (pI) and molecular weight (kDa) of the GRF proteins in the *S. spontaneum* were calculated using the online tool ExPASy (http://www.expasy.org/tools/). The subcellular location prediction of SsGRF proteins was carried out by the online tool WoLF PSORT (https://www.genscript.com/wolf-psort.html). A comparison of the GRF proteins present in *S. spontaneum* and *S. bicolor* was made using BioEdit v7.2.5 [42]. The conserved domains and motifs of the *S. spontaneum* GRF protein sequences were analyzed using the NCBI-CDD online portal and the MEME online program (http://meme-suite.org/tools/meme). The maximum number of motifs was set to 10, and the remaining parameters were default. A set of gff3 gene annotation files was used as input into TBtools v1.098 [43] to analyze the exon-intron structure of the *GRF* genes in *S. spontaneum*. The DNAMAN software 6.0 program was used to align multiple protein sequences of SsGRFs (Lynnon Biosoft, USA). The physical gene locations of GRFs in the *S. bicolor*, *Z. mays*, *O. sativa*, and *S. spontaneum* genomes were extracted from the genome annotation. The chromosomal distribution of all identified *S. spontaneum GRF* genes were mapped to *S. bicolor*, *Z. mays*, and *O. sativa* chromosomes using Mapchart software (Version 2.1) [44]. The circular map of syntenic analysis in the *S. spontaneum*, *S. bicolor*, *Z. mays*, and *O. sativa* genome was constructed using TBtools software v1.098 [43].

### Subcellular localization analysis

The SsGRF1 ORF was cloned and inserted into the *pCAMBIA1300-GFP* vector by infusion cloning. The *pCAMBIA1300-GFP* vector expresses an individual GFP was used as a control. The red fluorescent protein mKATE with nucleus localization signals (NLS, DPKKKRKV) [45], NLS-mKATE, were used as marker located in the nucleus. *N. benthamina* leaf infiltration was performed according to the protocol described [46]. Agrobacterium cells co-expressing SsGRF1-GFP and NLS-mKATE and those co-expressing GFP and NLS-mKATE were separately infiltrated into the two halves of a leaf. Leaves were harvested at 48 h postinoculation. Confocal images were acquired on a Zeiss LSM 800 microscope using a Plan-Apochromat 20×/0.8 M27 objective. The 488-, 561- and 640-nm lasers were used to excite GFP, mKATE and chlorophyll fluorescence, respectively. Emitted fluorescence was detected by GaAsP-Detector, set to detect 510 nm for GFP, 580 nm for mKATE and 685 nm for chlorophyll fluorescence.

### Gene expression analysis

Based on previous studies involving four groups of transcriptome data (different developmental stages and tissues, leaf gradient, circadian rhythm, and low-nitrogen (LN) stress), the expression patterns of *GRFs* in *Saccharum* were analyzed [47, 48]. As described previously, RNA preparation, cDNA library construction, and RNA-Seq library sequencing were performed [49, 50]. The transcriptome raw data were aligned to the reference gene model *S. spontaneum* AP85–441 using Trinity (https://github.com/trinityrnaseq/trinityrnaseq/wiki). Expression levels were calculated and normalized as fragments per kilobase million (FPKM) values as previously described [50, 51]. The heatmaps of gene expression levels were visualized using TBTools v1.098 [43] based on the transformed data of log_2_ (FPKM) values.

### Plant materials cultivation and treatments

To analyze expression patterns, two *Saccharum* species, *S. spontaneum* cultivar ‘SES-208’ (2 N = 8× = 64) and *S. officinarum* cultivar ‘LA-Purple’ (2 N = 8× = 64) were grown in the greenhouse of Fujian Agriculture and Forestry University. To study their expression profiles at multiple developmental stages, tissues samples including stems and leaves at the seedling stage, as well as leaf rolls, leaves, internode-3 (upper), internode-6 (central), and internode-9 (bottom) at the pre-mature and mature stages were collected as previously described [47]. To explore the expression profiles of leaf development, the second leaf of SES208 (11-day-old) and LA-Purple (15-day-old) was divided into 15 segments and 4 regions, leave samples including the basal zone (sink tissue), transitional zone (sink-source transition), maturing zone and mature zone (activated photosynthetic zone with full differentiation) were collected using a previously described procedure [52]. To analyze the expression profile of the circadian rhythm, leaves samples from mature plants of SES208 and LA-Purple were incessantly collected 12 times at a 2-hour interval in the first 24 h, followed by 7 times at a 4-hour interval in the next 24 h. The tissues were collected according to a method described previously between 6:00 a.m. on March 2, 2017, and 6:00 a.m. on March 4, 2017 [53].

To determine the expression pattern of sugarcane under low-nitrogen stress, two *Saccharum* hybrid cultivar, YT55 (LN-tolerant) and YT00-236 (LN-sensitive), belonging to sister lines were cultivated in sugarcane breeding bases (Wengyuan, Guangdong Province) of Institute of Nanfan & Seed Industry, Guangdong Academy of Sciences. Seedlings of 1-month-old YT55 and YT00-236 were transferred to a greenhouse with the condition of 20~28 °C temperature and 50~75% relative humidity in a normal nitrogen solution (7.5 mmol/L) for 20 days and then switched to a nitrogen-deficient solution (0.1 mmol/L) for starvation treatment according to a previous report [48]. Three biological replicates of the leaves and roots of half a dozen plants in individual pots were snap-frozen in liquid nitrogen at time points of 0 h, 6 h, 12 h, 24 h, 48 h, and 72 h after starvation and stored at -80 °C until further analysis.

### Validation of ***GRF*** gene expression levels by RT–qPCR analysis

The expression level of 2 *GRF* genes (*GRF1* and *GRF3*) was verified at 6 different time points (0 h, 6 h, 12 h, 24 h, 48 h, 72 h) in the leaves and roots of *Saccharum* hybrid varieties YT55 and YT00-236 under LN conditions by real-time quantitative PCR (RT-qPCR). The total RNA of the collected roots and leaves was extracted using RNAprep Pure Plant Plus Kit (Tiangen Biotech, China) according to the manufacturer’s instructions. The quality of RNA was evaluated by electrophoresis on a 1% agarose gel and scanned using a NanoDrop spectrophotometer (Thermo Scientific, USA). Reverse transcription qPCR and relative expression levels were implemented as previously described [50]. To normalize the expression levels, the constitutively expressed *eukaryotic elongation factor 1a* (*eEF-1a*) and *β-actin* gene were used as the reference gene [54]. Samples at 0 h were selected as control. The relative gene expression level of each gene was calculated using the 2^−ΔΔCt^ method [55]. A total of three biological and three technical replicates were performed for each sample. The primers for quantitative PCR analysis were designed using Primer Premier 5.0 (Premier Biosoft, USA). The primer sequences are cataloged in Table S5. A three-step PCR procedure was conducted with the aid of the 7500 Real-Time PCR System (Applied Biosystems, USA). Statistical analysis of relative expression was performed using IBM SPSS Statistics 26.0 software (IBM SPSS Inc., USA). After assessing the equality of variances by ANOVA, Duncan’s test was used for multiple comparisons. It was considered statistically significant when the *P* value was less than 0.05.

## Results

### Identification of ***SsGRF*** genes in ***S. spontaneum***

To identify *GRF* genes in sugarcane, BLASTp and Hidden Markov Model (HMM) searches were conducted. The 46 sequences from the 46 reported GRF proteins (Table S1, File S1), including those from *Arabidopsis* (9), rice (12), maize (15), and sorghum (10), were used as BLASTp queries to scan the wild sugarcane reference genome *S. spontaneum* AP85-441 [30]. Finally, a total of 10 *SsGRFs* were identified from the *S. spontaneum* genome without taking 24 redundant alleles (Table S2). We named *Saccharum GRF* genes as *SsGRF1* to *SsGRF10* following the naming rule proposed by Schilling et al. [33], the corresponding characteristics of the *SsGRF* family members are shown in Table 1. Full-length cDNA varied from 753 to 1758 bp, and their deduced protein products comprised 251 amino acids (aa) (SsGRF5) to 585 aa (SsGRF1), with the predicted molecular weights ranging from 26.00 kDa to 61.18 kDa. The predicted isoelectric points (pI) of the *SsGRFs* varied from 4.90 (SsGRF3) to 9.21 (SsGRF5). These *SsGRFs* were distributed on all eight chromosomes (Chr) except chr3 and chr 7 of *S. spontaneum*. According to subcellular localization predictions, all GRFs except SsGRF3 resided mainly in the nucleus, while SsGRF3 was located in the chloroplast, nucleus, and cytoplasm. The SsGRF proteins lacked transmembrane helical segments (TMHs) (Table S4). The TMH and subcellular localization of GRF proteins in *S. spontaneum* were the same as those of other representative species, which suggested that they might have the same functions (Table S4). The amino acid sequence alignment of SsGRFs with their orthologs in sorghum revealed that they share identities ranging from 88.6 to 95.3%, with a mean of 92.0% (Table 1). According to SsGRF protein sequence comparisons, SsGRF4 and SsGRF8 share the highest identity (53.9%), whereas SsGRF1 and SsGRF3 share the least identity (9.0%) (Table S1). The results indicate that the *GRF* gene family is highly conserved, but some members have shown obvious functional differentiation during evolution.

**Table 1:** The characteristics of *GRF* family members in Saccharum

Gene name	Gene ID	Chr^a^	CDS^b^	AA^c^	Mw^d^	pI^e^	PL^f^	***Sorghum bicolor***	***Sorghum*** ortholog ID	Similarity^g^
*SsGRF1*	Sspon.01G0026220-1A	1	1758	585	61.18	6.63	Nucl: 13	*SbGRF1*	Sobic.001G104500	94.5
*SsGRF2*	Sspon.01G0024960-1A	1	1245	415	45.48	9.20	Nucl: 13	*SbGRF2*	Sobic.001G139800	94.5
*SsGRF3*	Sspon.02G0007980-3D	2	780	259	27.39	4.90	Chlo: 6, Nucl: 4, Cyto: 2	*SbGRF3*	Sobic.002G297800	91.8
*SsGRF4*	Sspon.04G0023310-2C	4	1188	396	42.15	7.66	Nucl: 13	*SbGRF4*	Sobic.004G269900	92.3
*SsGRF5*	Sspon.04G0006300-1A	4	753	251	26.00	9.21	Nucl: 11, Cyto: 3	*SbGRF5*	Sobic.004G282601	89.0
*SsGRF6*	Sspon.08G0012220-1P	4	1191	397	43.05	8.61	Nucl: 12, Chlo: 1	*SbGRF6*	Sobic.004G317000	90.5
*SsGRF7*	Sspon.06G0015340-1A	6	969	323	34.54	8.67	Nucl: 13	*SbGRF7*	Sobic.005G150900	94.2
*SsGRF8*	Sspon.05G0023450-1B	5	1137	378	40.84	8.28	Nucl: 13	*SbGRF8*	Sobic.006G203400	88.6
*SsGRF9*	Sspon.08G0017060-1A	8	1221	406	43.63	8.45	Nucl: 9, Pero: 4	*SbGRF9*	Sobic.010G013500	95.3
*SsGRF10*	Sspon.08G0012220-1A	8	1053	351	37.81	9.20	Nucl: 12, Plas: 1	*SbGRF10*	Sobic.010G077200	89.4

*Nucl* Nucleus,*Chlo* Chloroplast, *Cyto* Cytoplasm, *Pero* peroxisome, *Plas* plasma membrane. Test k used for kNN is: 14.

^a^ Chromosomal position of the GRFs.

^b^ Length of the coding sequence in *GRF* genes.

^c^ Number of amino acids in GRF protein sequences.

^d^ Molecular weight (Mw, kDa) calculated by ExPASy (https://web.expasy.org/compute_pi/).

^e^ Isoelectric point (pI) predicted by ExPASy (https://web.expasy.org/compute_pi/).

^f^ Subcellular location of the GRF proteins predicted by WoLF PSORT (https://www.genscript.com/wolf-psort.html).

^g^ Protein sequence similarity (%) between sugarcane and sorghum calculated by BioEdit software.

### Subcellular localization of SsGRF1

**Fig. 1:**
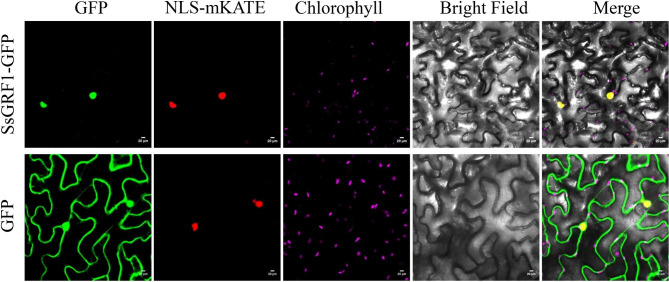
Subcellular location of SsGRF1 protein in *Nicotiana benthamiana* leaf epidermal cells. The SsGRF1-GFP or GFP was transiently co-expressed with the nuclear localization marker NLS-mKATE by *Agrobacterium*. Images of epidermal cells were captured using green fluorescence, mKATE fluorescence, chlorophyll fluorescence, visible light, and merged light. Confocal laser microscopy scanning was carried out 48 h after dark culture with a Zeiss LSM 800. Scale bars, 20 μm

To confirm the subcellular localization of the GRF, the ORF of SsGRF1 together with green fluorescent protein GFP were cloned and transiently expressed in tobacco leaf epidermal cells. An individual GFP was used as a control. Confocal scanning results showed that the SsGRF1-GFP fusion protein was present in the nucleus, while the GFP was distributed throughout the whole cells (Fig. 1). These results were in accordance with that in sequence predictions by the online tool WoLF PSORT, which indicated that SsGRF1 was mainly located in the nucleus.

### Phylogenetic and gene structure analysis

**Fig. 2:**
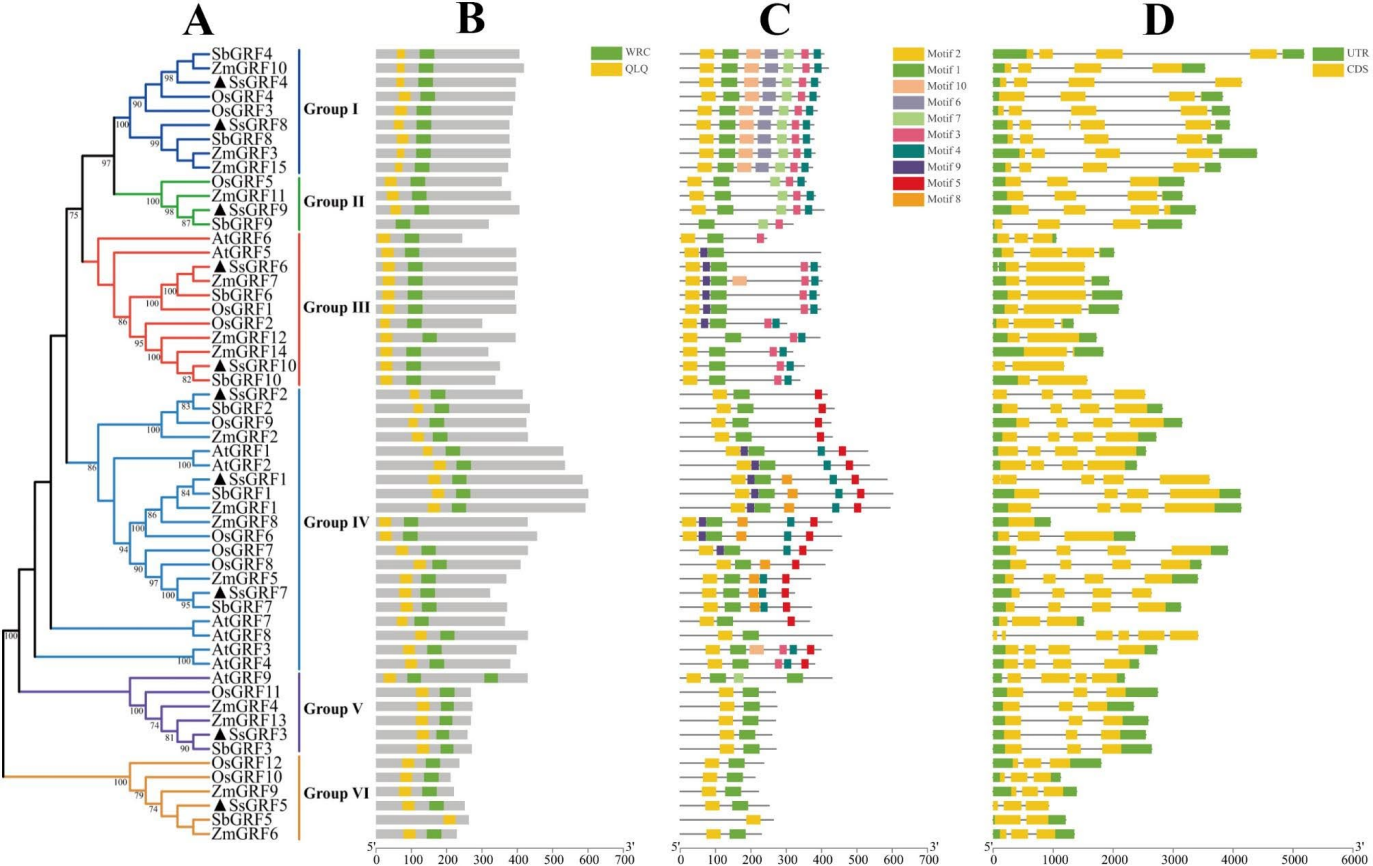
Phylogenetic tree of *GRF* gene family from sugarcane, sorghum, maize, rice, and *Arabidopsis* (**A**), as well as conserved domain (**B**), conserved motifs (**C**), and exon/intron organization (**D**). Phylogenetic tree was constructed using neighbor-joining methods and 1000 bootstrap replicates by MEGA X software. Black solid triangles were the new *GRFs* found in *Saccharum*, while bootstrap values lower than 70% were not shown. The *GRFs* were classified into six Groups (marked with different colored lines). The QLQ and WRC domains were highlighted by yellow and green boxes, respectively. There were 10 different motifs represented by different colored boxes in each of the *GRFs*. Exons and introns were denoted by yellow boxes and black lines, while untranslated (UTR) regions were represented by green boxes, respectively

To explore the evolutionary relationships of the *SsGRF* family members, 56 GRF protein sequences derived from *S. spontaneum*, *A. thaliana*, *S. bicolor*, *Z. mays*, and *O. sativa* were analyzed phylogenetically. The 10 SsGRF proteins were classified into six clades, herein referred to as Group I to VI, of which SsGRF4 and SsGRF8 belonged to Group I, SsGRF9 belonged to Group II, SsGRF6 and SsGRF10 belonged to Group III, SsGRF1, SsGRF2 and SsGRF7 belonged to Group IV, SsGRF3 belonged to Group V, and SsGRF5 belonged to Group VI (Fig. 2A). In all five species, Group IV was the largest (20 *GRFs*). In contrast, Group III and I comprised 11 and 9 *GRFs* each, whereas Group V, VI, and II included 6, 6, and 4 *GRFs* each (Fig. 2A). Similarly, in *S. spontaneum*, Group IV was the largest (3 *SsGRFs*), and Group I and III both comprised 2 *SsGRFs* each, while Group II, V, and VI all included 1 *SsGRFs* each (Fig. 2A). The 10 *GRF* genes in *S. spontaneum* were more closely related to those from *S. bicolor* than to those from the other three species, which was in line with a higher degree of protein homology between the two species (Table 1 and Table S1). Collectively, these studies suggested that *GRF* family members have evolved differently across various plants, and the SsGRF proteins exhibited a stronger relationship with SbGRF proteins than with any other GRF proteins.

**Fig. 3:**
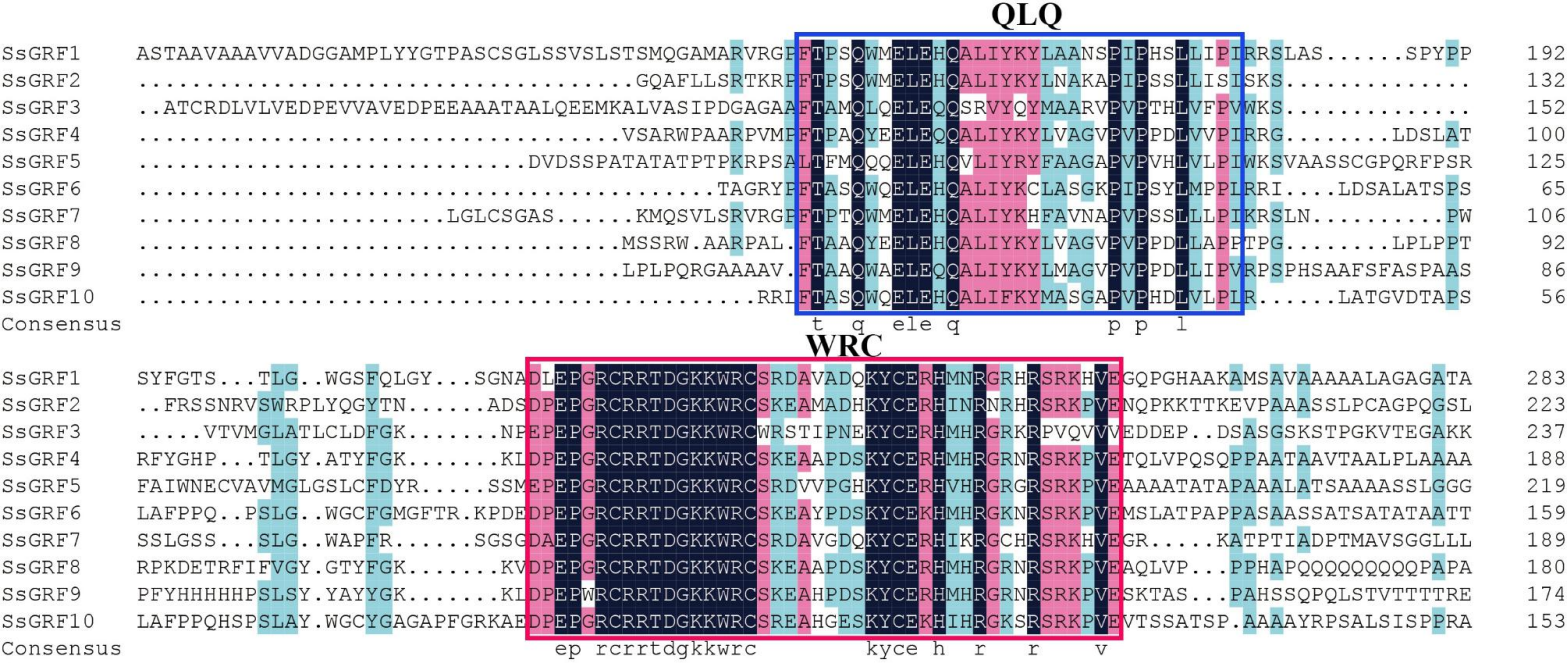
Amino acid sequence alignment of GRFs in *S. spontaneum*. Multiple alignments were conducted by DNAMAN. The numbers on the right side of the sequence indicate the position of amino acid residues, and the colors represent similarities in the protein sequences. The black shading box represents identical amino acid residues, and the red and blue shading boxes represent similar amino acid residues. As indicated by the boxes QLQ and WRC, the conserved domains are highly conserved in the GRF isozymes

The conserved domains of the GRF proteins are presented in Fig. 2B. The results indicated that all SsGRF proteins had both QLQ and WRC domains as do *A. thaliana*, *O. sativa*, *S. bicolor*, and *Z. mays* GRF proteins. Multiple sequence alignments of the QLQ and WRC domains in SsGRFs were performed to further understand the conserved characteristics of these two domains. The QLQ and WRC domains showed high conservation, whereas the amino acids in the WRC domain had greater conservation than those in QLQ (Fig. 3). The WRC domain contained 22 highly conserved amino acids (E3P4, R6C7R8R9T10D11G12K13K14W15R16C17, K26Y27C28E29, H31, R34, R38, and V43), whereas the QLQ domain contained only 9 highly conserved amino acids (T2, Q5, E8L9E10, Q12, P24, P26, and L29). In addition, the WRC domain of all SsGRF proteins also contained a zinc finger motif consisting of three cysteines and one histidine (Fig. 3) [56].

We further examined conserved motifs in the deduced SsGRF proteins using the MEME online program and detected a total of 10 motifs (numbered from motif 1 to motif 10) (Fig. 2C). As speculated, motif 1 and motif 2, corresponding to QLQ and WRC domains, were displayed across all SsGRF proteins. The same motif was consistently present in SsGRF proteins within the same group. For example, motifs 3 and 4 were presented only in Group III, while motifs 3, 4, 6, 7, and 10 only existed in Group I. These particular motifs may conduce to the complexity of function of *GRF* genes from diverse Groups.

To clarify the evolution of the *SsGRF* gene family, the exon-intron structures were analyzed (Fig. 1D). The number of exons of *SsGRF* genes ranged from 2 to 4. Most Group II, V, and VI genes had 3 exons, whereas Group I and V genes contained 4 exons. And Group IV genes comprised 2 exons. The number of exons for the same group of SsGRF genes was relatively constant. These findings reflect the structural similarity among the *S. spontaneum GRF* genes as well as the gain and loss of exons during evolution.

In summary, *GRF* genes from the same group were found to have conservative motifs and homologous exon-intron structures, which combined with phylogenetic analysis helped to maintain their phylogenetic relationships.

### Synteny analysis and gene duplication prediction

**Fig. 4:**
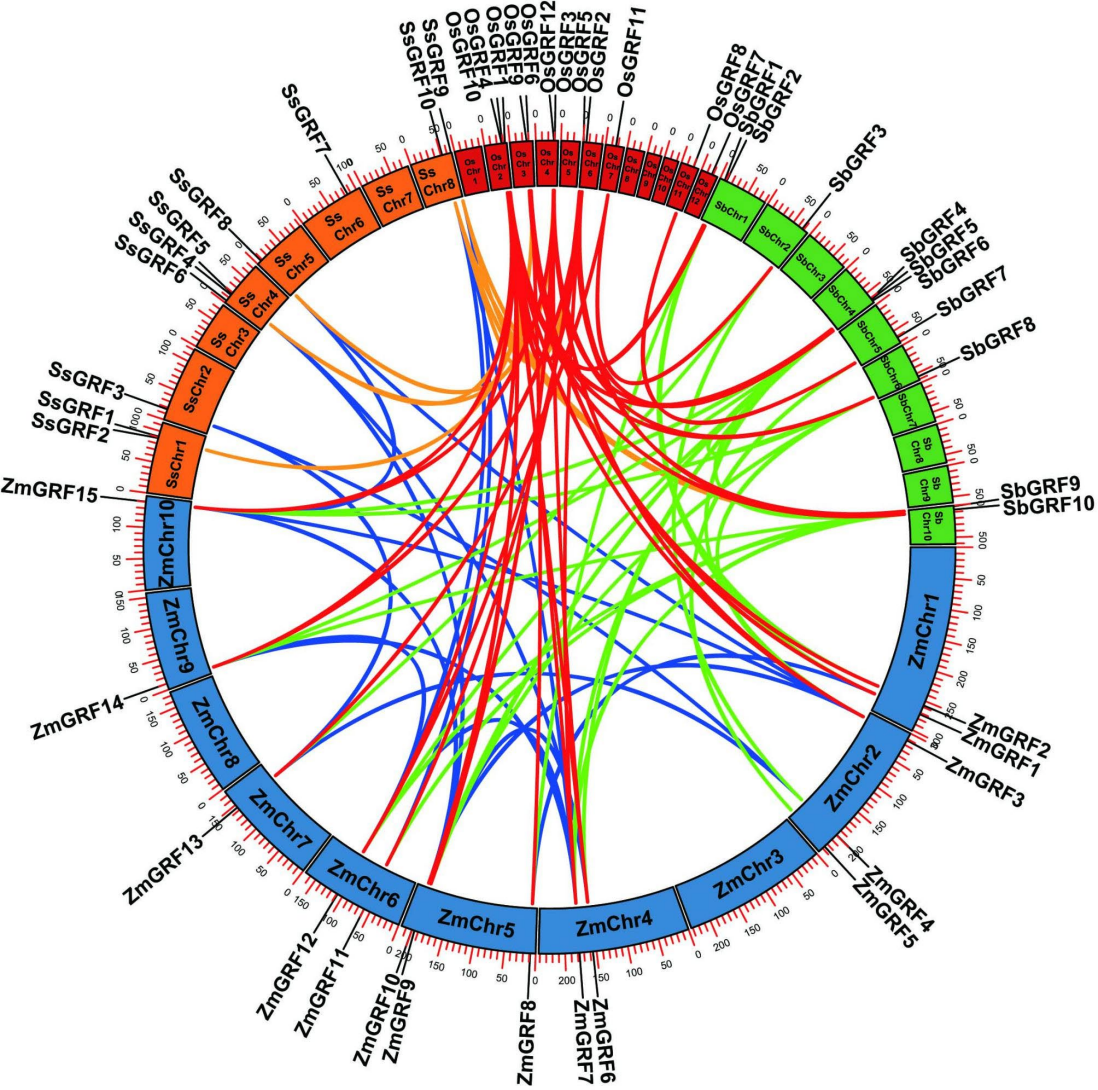
The location and collinearity relationships of *GRF* genes from sugarcane, sorghum, maize, and rice. Gene pairs of *GRFs* were mapped to their respective loci in a circular diagram. The chromosomes of sugarcane, sorghum, maize, and rice are indicated by boxes of various colors with the prefixes ‘Ss’, ‘Sb’, ‘Zm’, and ‘Os’, respectively. A series of numbers below each box represents the length of the corresponding chromosome in megabases. Different colored lines indicate duplications of the *GRF* genes

The synteny among *GRF* orthologous pairs of *S. spontaneum, O. sativa*, *S. bicolor*, and *Z. mays* were identified by comparative analysis to investigate the origin and evolutionary history of the *GRFs.* Identification of orthologous genes to *GRF* genes in closely related plants can help predict *GRF* gene function in sugarcane. Synteny relationships were found among 95 pairs of orthologous genes among the four species, including 8 pairs relating *S. spontaneum* and *S. bicolor*, 9 pairs relating *S. spontaneum* and *Z. mays*, 7 pairs relating *S. spontaneum* and *O. sativa*, 21 pairs relating *S. bicolor* and *Z. mays*, 15 pairs relating *S. bicolor* and *O. sativa*, 21 pairs relating *Z. mays* and *O. sativa*, and 0, 3, 2, 9 of intragenomic pairs among four species, respectively (Table S3 and Fig. 4). No species-specific syntenic relationship was observed in *S. spontaneum*. Three *SsGRFs* (*SsGRF4*, *SsGRF6*, and *SsGRF7*) were not mapped on any other *GRFs*. It was found that *S. spontaneum* had the least orthologous gene pairs among the four species, indicating that the *GRFs* were less conserved in *S. spontaneum* than in the other three species under evolutionary dynamics.

The *SsGRF* genes were unevenly distributed on the chromosomes in *S. spontaneum* based on the gff3 genome reference files (Table 1; Fig. 4). In *S. spontaneum*, the 10 *SsGRF* genes were distributed on six chromosomes (Chr), of which chromosomes (Chr) 4 had the most *SsGRF* genes (*SsGRF4*, *SsGRF5*, and *SsGRF6*), followed by chr 1, and chr 8, which contained 2 *SsGRF* genes (*SsGRF1/ SsGRF2*, *SsGRF9*/*SsGRF10*), respectively, the rest chr 2, chr 5, and chr 6 contained 1 *SsGRF* gene (*SsGRF3*, *SsGRF8*, and *SsGRF7*). The chromosomes distribution analysis indicated the *GRF* genes were most abundant on chr 4 in all examined *S. spontaneum*, possibly because of gene duplication events.

A distinction has been made between two types of gene duplication events among the four species above to gain a deeper understanding of their relationship to *GRF* genes (Table S4). Among the *SsGRFs*, 5 out of 10 genes (50.0%) were labeled as whole-genome duplication (WGD) or segmental duplication genes, while the rest (50.0%) were classified as dispersed duplicates. Similarly, the other three species *GRFs* were mostly classified as WGD or segmental duplication genes (24 out of 37, 64.9%), and the remaining *GRFs* were classified into dispersed duplicates (13 out of 37, 35.1%). In *O. sativa*, genes involved in WGD or segmental duplication events, as well as dispersed genes, accounted for 50.0% each (6 out of 12). In *S. bicolor*, genes involved in WGD or segmental duplication events, as well as dispersed genes, accounted for 40.0% (4 out of 10) and 60.0%, respectively. While in *Z. mays*, genes involved in WGD or segmental duplication events, as well as dispersed genes, accounted for 93.3% (14 out of 15) and 6.7%, respectively. These results showed that WGD or segmental duplication and dispersed duplication were the main force driving the expansion of the *GRF* gene family.

Functional divergence and evolution can result from gene duplication events. To determine the selection pressure associated with the duplication of *GRF* gene pairs within species, the ratio of nonsynonymous substitution rate (Ka) to the synonymous substitution rate (Ks) was calculated. The orthologous *GRF* genes were identified between *S. spontaneum* and *S. bicolor*. In *S. spontaneum* and *S. bicolor*, the Ka/Ks ratios of all gene pairs were less than one (Table 2), suggesting that purifying selection was the primary driving force for their evolution. Based on the Ks value, the divergence times for the paralogous pairs of *SsGRFs* and their orthologous pairs of *SbGRFs* were calculated (Table 2). In terms of divergence time, *S. spontaneum* diverged from *S. bicolor* 7.779 million years ago (Mya) [57]. In the present study, *SsGRF1* and *SsGRF7* diverged with their orthologous *SbGRFs* at 6.717 Mya and 7.427 Mya, respectively, which were shorter than those of *S. spontaneum* and *S. bicolor* (7.779 Mya). As a comparison, the remaining 8 *SsGRFs* diverged with their orthologs at 7,848 Mya and 14,403 Mya, respectively, which were longer than those of *S. spontaneum* and *S. bicolor*.

**Table 2:** Nonsynonymous (Ka) and synonymous (Ks) substitution rates and estimated divergence time for paralogous *GRF* genes in *Saccharum* and sorghum

Paralogous pairs	Ka	Ks	Ka/Ks	Divergence time (Mya)
*SsGRF1* vs. *SbGRF1*	0.013	0.082	0.159	6.717
*SsGRF2* vs. *SbGRF2*	0.020	0.096	0.210	7.848
*SsGRF3* vs. *SbGRF3*	0.017	0.120	0.143	9.852
*SsGRF4* vs. *SbGRF4*	0.027	0.110	0.249	9.003
*SsGRF5* vs. *SbGRF5*	0.066	0.134	0.487	11.023
*SsGRF6* vs. *SbGRF6*	0.025	0.176	0.142	14.403
*SsGRF7* vs. *SbGRF7*	0.015	0.091	0.167	7.427
*SsGRF8* vs. *SbGRF8*	0.031	0.105	0.297	8.595
*SsGRF9* vs. *SbGRF9*	0.014	0.121	0.119	9.953
*SsGRF10* vs. *SbGRF10*	0.040	0.131	0.309	10.714

### Expression patterns of ***GRF*** genes during various plant developmental stages and in different tissues

**Fig. 5:**
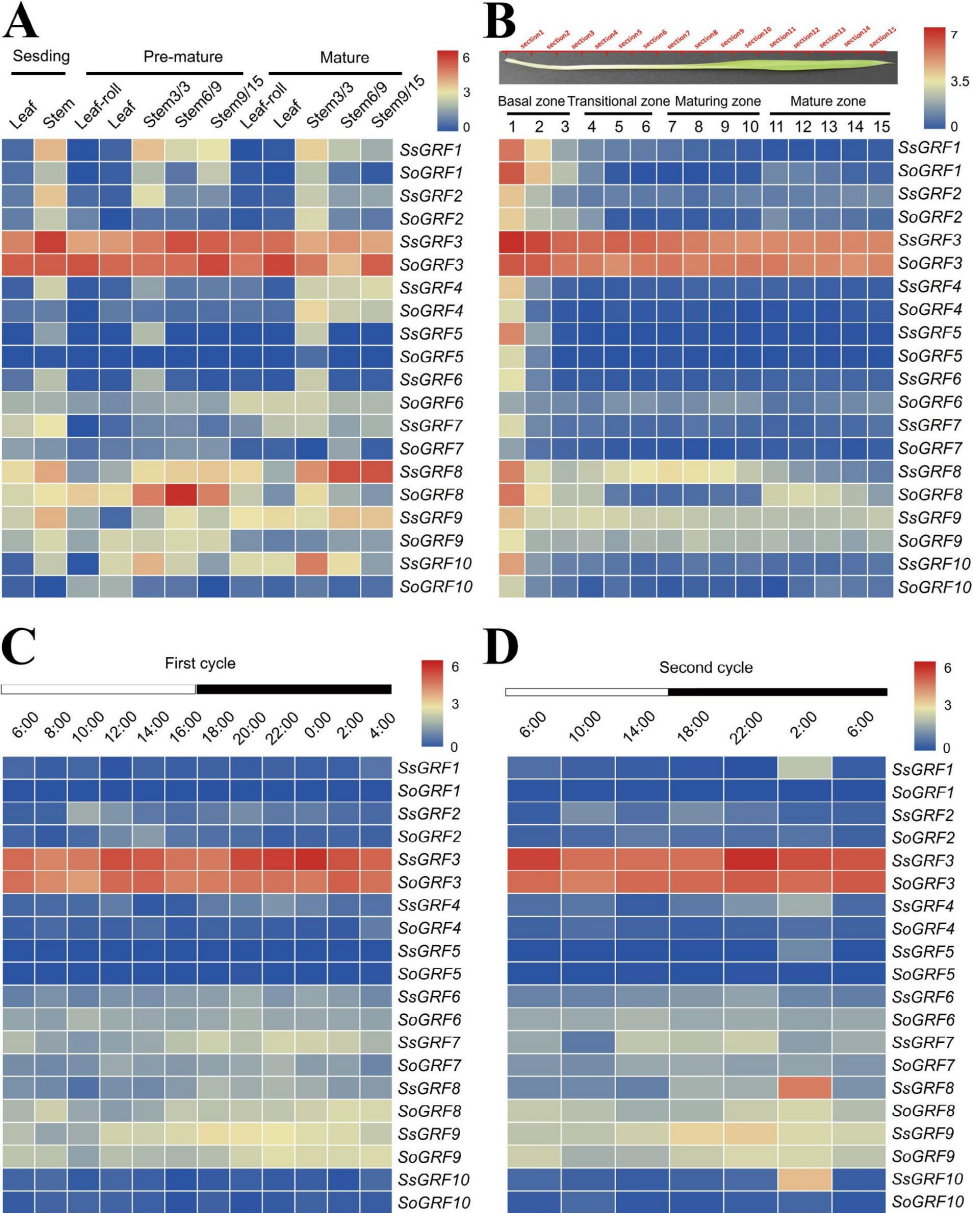
The expression pattern of *GRF* genes during various developmental stages and different tissues in *S. spontaneum* and *S. officinarum* based on log2-transformed FPKM values. (**A**) Heatmap based on gene expression in different tissues at various stages. (**B**) Heatmap based on gene expression across leaf gradients. **C**&**D**) Heatmap based on gene expression during the diurnal cycles. The heatmap was plotted using the TBtools software (v1.098). The scale bar represents the log2 normalized expression values after normalizing expression to genes using the average linkage algorithm. The red color represents higher expression, while the blue color indicates lower expression

To explore the expression patterns of *GRF* genes in multiple plant growth and development processes, we investigated the expression pattern of *Saccharum GRF* genes during the development stages. Expression patterns of *GRFs* between 2 *Saccharum* species, *S. spontaneum* and *S. officinarum*, were analyzed using available transcriptome data [47] during 3 developmental stages in various tissues (Fig. 5A). The expression levels varied among genes, with some genes exhibiting tissue-specific expression. Among the 10 *GRF* genes analyzed, 1 gene (*GRF3*) was relatively highly expressed in all developmental stages and tissues, reflecting its overall involvement in *Saccharum* plant development; whereas 5 genes (*GRF2/4/5/7/10*) exhibited relatively low or barely detectable expression levels in all examined tissues in different growth stages. Additionally, almost all *GRFs* were expressed at higher expression levels in stems than in leaves. Notably, 2 genes (*GRF1* and *GRF10*) showed higher expression levels in *S. spontaneum* than in *S. officinarum*, while 2 genes (*GRF3* and *GRF6*) were expressed equally in 2 *Saccharum* species. In addition, *GRF8* showed higher expression levels in *S. spontaneum* than in *S. officinarum* at the seedling and mature stage but showed lower expression levels in *S. spontaneum* than in *S. officinarum* at the pre-mature stage. The results presented here suggested that *GRF* genes function differently at various developmental stages and may affect biological processes in different tissues. To confirm this, detailed analyses of their expression in roots, meristematic, and reproductive tissues are needed for a more complete understanding of their functions.

The study of the expression pattern of *GRF* genes in continuously developing leaf segment gradients from *S. spontaneum* and *S. officinarum* provided further insights into the functional divergence of *GRF* genes for photosynthesis and sugar transport in source tissues (Fig. 5B). Similar to the expression pattern at various developmental stages, *GRF3* were relatively highly expressed in all leaf segments, indicating their overall involvement in *Saccharum* photosynthesis and sugar transport; whereas 5 genes (*GRF2/4/5/7/10*) showed low or undetectable expression levels, suggesting their limited contribution to photosynthesis and sugar transport. Interestingly, almost all *GRFs* declined gradually from the basal zone to the mature zone in *S. spontaneum*, while these genes decreased from the basal zone to the transition zone but increased from the maturing zone to the mature zone in *S. officinarum*. Notably, *GRF3* and *GRF9* showed higher expression levels in *S. spontaneum* than in *S. officinarum in* the basal zone and the transition zone but expressed equally in the maturing zone and the mature zone. In addition, *GRF1* and *GRF8* showed lower expression levels in *S. spontaneum* than in *S. officinarum* at the basal zone, while *GRF6* showed the opposite at the basal zone. Interestingly, these results indicated functional divergence of the *GRF* genes in leaf segment gradients, and interspecies differentiation could also contribute to this divergence.

To examine the expression patterns of *GRFs* during diurnal cycles, we examined the expression pattern of the mature leaves in the 2 *Saccharum* species over a 24 h period at 2 h intervals followed by 4 h intervals over another 24 h (Fig. 5C and 5D). Similarly, the transcriptome profiles at different developmental stages as well as in the leaf segment gradient, *GRF3* were relatively highly expressed at all time, whereas 5 genes (*GRF1/2/4/5/10*) showed either very low or undetectable expression levels in all examined leaf segments, further supporting their involvement or limited roles in growth and development. Additionally, 3 genes (*GRF3/7/8*) were expressed higher at night than in the daytime in 2 *Saccharum* species, whereas *GRF9* was highest in the evening and constitutively expressed at other times in *S. spontaneum* or *S. officinarum*. Notably, 3 genes (*GRF3/7/9*) showed higher expression levels in *S. spontaneum* than in *S. officinarum*. These findings suggested the functional divergence of the *GRF* genes in diurnal rhythms.

By analyzing the expression pattern of GRF genes in 2 *Saccharum* species at various developmental stages in various tissues, leaf segment gradients, and diurnal rhythms (Fig. 5), we found that *GRF3* maintained a high degree of expression in each period, and *GRF2/4/5/10* expressed weakly in each period, whereas other *GRF* genes displayed distinct expression patterns in different period. These results may provide valuable information on the role of these genes in sugarcane growth and development.

### Expression patterns of ***GRF*** genes under low-nitrogen stress

**Fig. 6:**
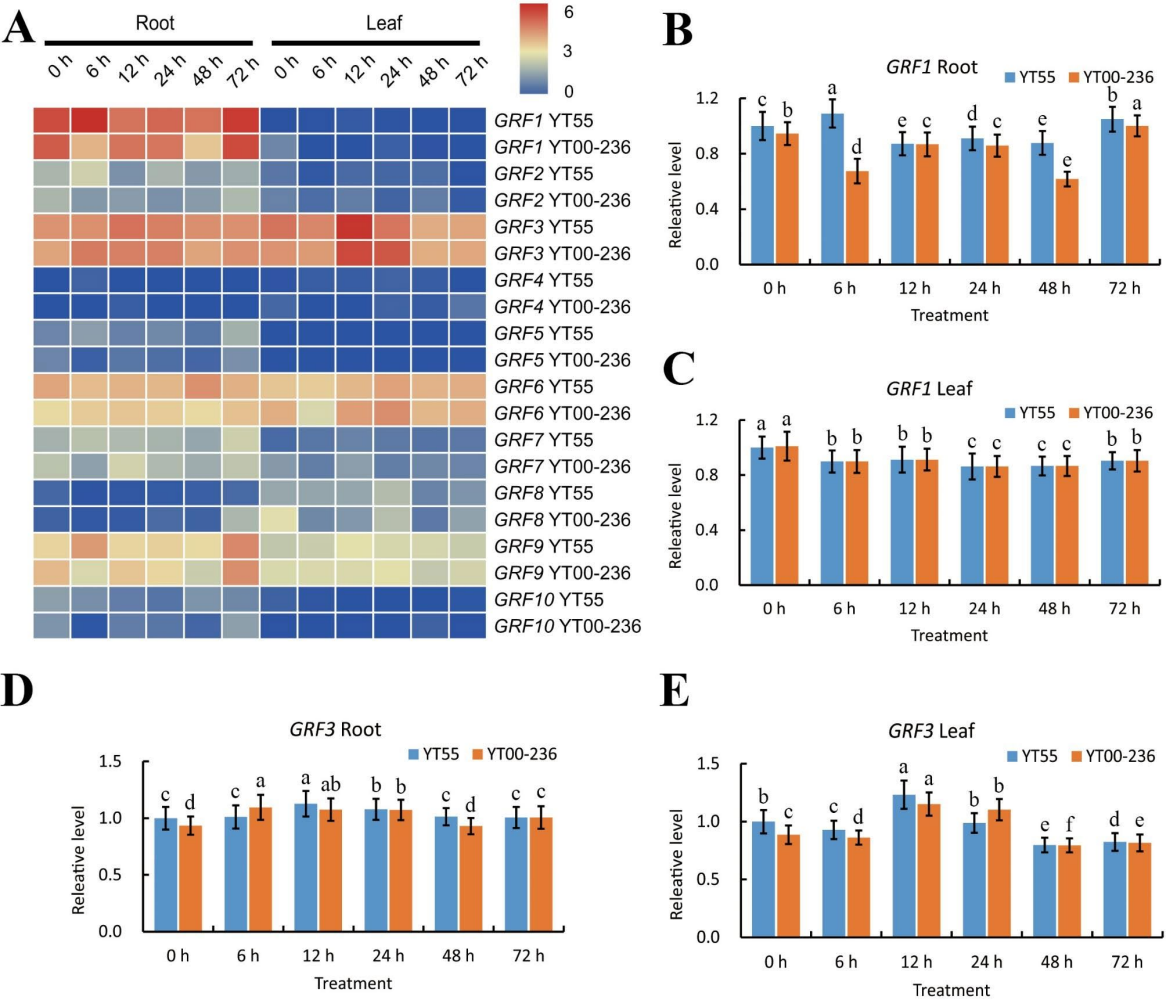
The expression pattern of *GRF* genes in *Saccharum* hybrid YT55 and YT00-236 based on log2-transformed FPKM values (**A**) under low-nitrogen stress conditions and verification of *GRF1* and *GRF3* expressions in root and leaf by RT-qPCR (**B**, **C**, **D**, and **E**). Seedlings of TY55 and TY00-236 were subjected to a nitrogen treatment of 100 mM, and samples were taken at 0, 6, 12, 24, 48, and 72 h after the treatment. The expression at 0 h was set to 1.0. Values were mean ± SD of three replicates. Bars with different letters were significantly different at the p < 0.05 level

To study the functional divergence of *GRF* genes in response to low-nitrogen (LN) stress in sugarcane, we investigated the expression patterns of *Saccharum GRFs* under LN stress (Fig. 6A). The *GRF* family genes in roots and leaves from 2 *Saccharum* hybrid varieties YT55 and YT00-236 at 0 h, 6 h, 12 h, 24 h, 48 h, and 72 h exhibited different expression patterns. Among the 10 *GRF* genes analyzed, *GRF3* were relatively highly expressed in both roots and leaves in the 2 *Saccharum* hybrid varieties, whereas 5 genes (*GRF2/4/5/8/10*) demonstrated considerably low or undetected expression levels in all LN stress, suggesting their involvement or limited roles in abiotic stress. Notably, in the roots of YT55, *GRF1* and *GRF9* were up-regulated within 6 h, thereafter, down-regulated at 12 h, but were then re-regulated at 48 h and reached a stably high level at 72 h, while in the roots of YT00-236, they down-regulated within 6 h, up-regulated at 12 h, then up-regulated at 48 h, and finally reached a high level at 72 h. In addition, *GRF3* was constitutive expressed in the roots of YT55 and YT00-236, while in the leaves, the level decreased within 6 h, then went up within 12 h, then went down again within 24 h, and finally remained low for 72 h after that. Importantly, *GRF1*, *GRF7*, and *GRF9* showed higher expression levels in roots than in leaves, while *GRF3* was the opposite. Intriguingly, in the roots of YT55, *GRF1* and *GRF6* expressed higher than those of YT00-236. These results may elucidate the discrepancy in LN tolerance between 2 *Saccharum* hybrid varieties. To explicitly test the reliability of transcriptome data, we ulteriorly analyzed the relative expression level of *GRF1* and *GRF3* in 2 *Saccharum* hybrid varieties at 0 h, 6 h, 12 h, 24 h, 48 h, and 72 h under LN stress by RT-qPCR method (Fig. 6B, 6C, 6D, and 6E). The findings indicated that the expression level of *GRF* genes tested by RT-qPCR was directly correlated with the transcriptome data, suggesting that the transcriptome data was believable and may offer alternative genes for cultivating stress-tolerant cultivars of sugarcane.

## Discussion

Growth-regulating factors (GRFs) are transcription factors specific to plants that are selected mainly for crop genetic improvement and which are important regulators of plant growth and development [58]. In recent years, the function and evolution of *GRF* members of various plants have been analyzed with a systematic bioinformatics method [5–7, 9, 49]. Sugarcane (*Saccharum* spp.) is an important crop around the world and provides essential sugar and energy for daily life [59]. However, little is known about the identities and expression profiles of its *GRF* genes. In the present study, 10 *SsGRFs* were identified in the genome database of wild sugarcane *S. spontaneum*. Characterization of the phylogeny, gene structure, synteny, gene duplication, and expression patterns of the *GRF* gene family in *Saccharum* was performed to investigate their evolution and potential functional differentiation.

Evolutionary history analyzes of the *GRF* family in various species help to speculate on gene function [3]. According to the analysis of the phylogenetic relationships, the *Saccharum GRF* family members were categorized into six groups. Research has shown that *GRF* members of rice, maize, *A. thaliana*, and rapeseed can be classified into three to six groups [60]. In the present study, phylogenetic analysis of *GRFs* of *S. spontaneum*, *A. thaliana*, *O. sativa*, *S. bicolor*, and *Z. mays* was compared. It was revealed that most *SsGRFs* priority clustered with *GRFs* in *S. bicolor*, followed by *Z. mays*, *O. sativa*, and *A. thaliana*. The results indicated that the *GRFs* in sugarcane were strongly associated with those in *S. bicolor*. The number of *GRF* genes in Group IV is greater than in other Groups, suggesting that the variability in the number of *GRF* genes in the various groups may be the result of individual gene gain or loss in these groups. Our results displayed that *GRFs* clustering in the same Groups shared similar gene structures, and oppositely, *GRFs* in various Groups were highly diversified, continuing to prove the genetic conservation within the *GRF* gene family. The same results have been reported with a variety of plants including *Arabidopsis*, rice, poplar, grape, and soybean [2, 6, 8, 9]. Multiple alignment analysis demonstrated that WRC domains were more conserved than QLQ domains and the two domains were highly homologous in the different *GRF* genes, indicating that *GRF* genes of different plant species might share a common ancestor. which were in agreement with previous studies [9, 60]. The gathered results support the fact that the protein encoded by the *SsGRF* gene family has evolved similarly to proteins encoded by other genes in crops.

The localization of proteins in diverse organelles may correlate with their function [61]. Previous reports have indicated that the nuclear localization signal (NLS) plays a crucial role in locating GRF protein in the nucleus [8], which was supported by our results on the subcellular location of GRFs in sugarcane, sorghum, maize, and rice (Table 4S). The protein sequences of SsGRF were blasted, and the results indicated similarities within the members were 9.0 ~ 53.9% and shared 88.6–95.3% homology with *S. bicolor* (Table S1), respectively. The findings of this study were in agreement with those of earlier studies on *A. thaliana* [8] and other higher plants [60, 62], suggesting high differentiation among the members of the *GRF* gene family and great conservation among the same type of *GRFs*.

Gene duplication is the primary mechanism for generating evolutionary innovations as well as a key factor in gene family evolution, such as whole-genome duplication (WGD) / segmental duplication and tandem duplication [63, 64]. Repeated episodes of tandem duplication and segmental duplication (or WGD) events are two major types of gene duplication events during the evolution of the plant genome [65]. Segmental duplication/WGD is a large-scale duplication event that leads to an amplification of a gene family [64]. The *Saccharum* genome has undergone two WGD events, which were directly responsible for most of the expansion of numerous gene families [30]. The present results indicated that WGD/segmental duplication and dispersed duplication significantly contributed to the expansion of the *GRF* gene family in *S. spontaneum*, and none of the *SsGRF* genes were detected to be tandem duplication (Table S4). There has been prior research on this phenomenon [66]. Gene family duplication patterns across species may exhibit a similar nonrandom origin pattern [67]. In this study, *S. spontaneum* and *O. sativa* were primarily driven by dispersed duplication, while *Z. mays* was primarily driven by WGD/segmental duplication. (Table S4). Based on these results, the primary duplication patterns of the *Saccharum GRF* gene family were not always severely conserved, and nonrandom patterns appeared to be consistent from diverse sources, which were following the previously reported expansion of the *GRF* gene family in other plant species [66]. The ancestor of *S. officinarum*, *S. spontaneum*, diverged about 7.779 Mya from sorghum, whereas *S. spontaneum* diverged about 769 thousand years ago from *S. officinarum* [57]. In the current study, the divergence times of two out of 10 *SsGRFs* with their orthologues *S. bicolor GRFs* (*SbGRFs*) were shorter than 7.779 Mya (Table 2), while the rest 8 of 10 *SsGRFs* were longer than 7.779 Mya. Based on these findings, the duplication of *SsGRFs* with their orthologs most likely occurred after the divergence of *S. spontaneum* and sorghum. The Ka/Ks ratios between paralogous pairs of *SsGRFs* and their orthologous *SbGRFs* were calculated (Table 2). In general, Ka/Ks ratio less than and more than 1 indicates purifying and positive selection pressures, respectively. A Ka/Ks ratio equal to 1 signifies neutral selection. All Ka/Ks ratios of *SsGRFs* were less than 1, suggesting that the evolution of *GRF* genes in sugarcane was influenced by strong purifying selection. The purifying selection pressure may help sustain the conserved structures of *SsGRF* genes during evolution.

The study of gene expression patterns helps researchers to better understand plant species’ biological properties, and previous reports concluded that *GRF* genes played multifunctional roles in plant growth and developmental processes [68]. More and more evidence has indicated that *GRFs* perform particular gene expression patterns associated with their function in different species. In *Arabidopsis*, *AtGRF1* and *AtGRF3* were expressed at a high level in the roots [22]. In rice, *OsGRF10* was expressed at a high level in the leaves [6]. *OsGRF4* controlled seed size by facilitating cell division and cell proliferation [20]. *OsGRF6 was* involved in adjusting the rice number of grains/spike [21]. The overexpression of *OsGRF10* in transgenic rice plants showed fewer tillers [69]. The *BnGRF2a* can notably improve the weight and oil content of transgenic rapeseed seeds [19]. The expression level of almost all *GRFs* in *Saccharum* was expressed higher in immature tissues than in mature tissues, suggesting that they might play a part in regulating plant development, which is associated with previously reported functional roles of *GRF* genes [9, 70]. During different stages of vegetative growth, *GRF3* expression was mainly detected, indicating the importance of gene function and its conservation. According to the transcription levels of *GRF* genes in various tissues in 2 *Saccharum* species and previous studies, *GRF* genes may play an important role in developing immature tissues in sugarcane, and their function needs to be investigated further.

Plants have evolved a range of signal pathways and defensive systems to resist stresses. In previous studies, the activation of gene response stresses improved the plant’s resistance [71]. Amounts of evidence have indicated that the *GRF* genes were responsive to abiotic stress, and alterations in their expression can enhance crop response to adverse conditions [1, 2]. The over-expression of *AtGRF7* in *Arabidopsis* under stress conditions improved tolerance to salt and drought stress [1]. Despite some researches having revealed that the *GRF* gene plays a crucial role in abiotic stress responses, we still have no idea if members of the *Saccharum GRF* family do as well respond to nutrition stress. Nutrition stress intensely affects plant growth and productivity. In this study, the expression levels of *Saccharum GRF* genes varied to different degrees in response to low-nitrogen. Gene expression patterns can offer significant clues for gene function, and the RT-qPCR verification of two selected *GRFs* show tissue-specific expression patterns in leaves and roots (Fig. 6). Numerous experiments have been conducted on YT55 and YT00-236 concerning nitrogen utilization and regulation [72, 73]. The nitrogen utilization of these two varieties was detected by analyzing the physiological and morphological indicators such as nitrogen and dry matter content, and root phenotype. There was an observable difference in all the indicators of YT55 and YT00-236, indicating that YT55 had a higher NUE than YT00-236 [72, 73]. Nevertheless, there was no clear explanation for the NUE differentiation between YT55 and YT00-236. In the current study, the expression level of *GRF1* in roots was higher than in leaves, while *GRF3* was the opposite in 2 *Saccharum* hybrid varieties. In addition, the expression level of *GRF1* in roots of YT55 was higher than those of YT00-236, while *GRF3* was quite different. There is a difference in NUE between YT55 and YT00-236 due to transcription patterns of *GRF1* and *GRF3* in the low-nitrogen response. Since only a few studies have explored the role of *GRF* genes in nutrition stress, this work provides new genetic resources for further research on the functions of *GRF* gene family members in different abiotic stress tolerance and then used to cultivate resistant sugarcane varieties.

## Conclusion

In the present study, we identified 10 *SsGRF* genes in the genome of wild sugarcane and analyzed their expression patterns under normal growth and low-nitrogen stress conditions. Phylogenetic analysis indicated that SsGRF proteins were categorized into six groups, and similarly structured and conserved motifs further demonstrated the similarity of members within the same group. Ka/Ks analysis suggested that *GRF* genes experienced strong purifying selection during evolution. The expression patterns of *SsGRFs* in various tissues indicated that *SsGRFs* may have diverse regulatory roles connected with the growth and development of *Saccharum* species. The RT-qPCR verification of *SsGRF1* and *SsGRF3* expression under low-nitrogen stress demonstrated that they may affect abiotic stress resistance by modulating certain stress-responsive. Taken together, the data generated in this study may offer precious resources for further investigating the function of *Saccharum GRF* genes, especially regarding diverse developmental stages and abiotic stress responses, which will promote their application in cultivated sugarcane breeding.

## Electronic supplementary material

Below is the link to the electronic supplementary material.

Supplementary Material 1

Supplementary Material 2

Supplementary Material 3

Supplementary Material 4

Supplementary Material 5

Supplementary Material 6

## Data Availability

All RNA-seq data can be downloaded from the sugarcane database website (http://sugarcane.zhangjisenlab.cn/sgd/html/index.html). The *S. spontaneum* genome project was deposited into Genbank with accession numbers: QVOL00000000.

## Declarations

### Authors’ information

^1^Guangdong Sugarcane Genetic Improvement Engineering Centre, Institute of Nanfan & Seed Industry, Guangdong Academy of Sciences, Guangzhou 510316, Guangdong, China. ^2^Zhanjiang Research Center, Institute of Nanfan & Seed Industry, Guangdong Academy of Sciences, Zhanjiang 524300, Guangdong, China.
