# Supplementary material for: Genome-wide characterization and expression analysis of the growth-regulating factor family in Saccharum
Source: BMC Plant Biol. 2022 Nov 2;22:510. doi: 10.1186/s12870-022-03891-4 (PMC9628180; doi:10.1186/s12870-022-03891-4)
Supplement: Supplementary file 1 — Supplementary Material 1 [file 12870_2022_3891_MOESM1_ESM.doc]

>SsGRF1_Sspon.01G0026220-1A

MDLMGGVVMEAGGGGASELGLLGGGSSSRLLKHGRGNADHGWGGGGRAKQARTTASVVAGDVVEAAKAAAPFLLGSCSPGHGGEQMLSFSSAAAAASSCASTAAVAAAVVADGGAMPLYYGTPASCSGLSSVSLSTSMQGAMARVRGPFTPSQWMELEHQALIYKYLAANSPIPHSLLIPIRRSLASSPYPPSYFGTSTLGWGSFQLGYSGNADLEPGRCRRTDGKKWRCSRDAVADQKYCERHMNRGRHRSRKHVEGQPGHAAKAMSAVAAAAALAGAGATAAGLTVNQHQQPAKSYATGATEPCSLQYNRELVNKQNESENMQDSDNLSMLTSMTTGNTGSVFPLSKQNNPFEVTSSRPEFCLVSSDSLMSSPHSSLENVNLLSSQSLNEHQSSASLQHFVDWPRTPAQGGLSWPEAEDMQAQRSQLSISAPMASSELSSASTSPIHEKLMLSPLKLSREYSPTGFSIAANRDEASQLEATWATMFRDSSMGGPLGEVLTKNGNVEAKNCLSAPLNLLTDYWDSSHGMESSPVGVLQKTAFGSVSSSTGSSPRIESHGAYDGISNLRDDLGSIVVSHPSIRLV

>SsGRF2_Sspon.01G0024960-1A

MSAEFCAAAGMELGVGDVMGLQQGIAITAPSPRGSGDLGLLKRAALTQAAAGPYPSPFLDEQKMLRFSKAAHTLPSGLDFGGPSGQAFLLSRTKRPFTPSQWMELEHQALIYKYLNAKAPIPSSLLISISKSFRSSNRVSWRPLYQGYTNADSDPEPGRCRRTDGKKWRCSKEAMADHKYCERHINRNRHRSRKPVENQPKKTTKEVPAAASSLPCAGPQGSLKKAKVNDSKPGTVSCWTDSLNRTMLSREKANKPTEDNSLLLNSTNSQPTLSLLSQLKQQNKPDKLGPTLENESNSDTILKAWGGNQPSHKSISSTQHHDAESLHSVLQNFSLAQNEKMESEKNKYSDSMLVSSTFYSADGPRSTCLTPNMTQVQQDCISSSWEMPQGGPLGEILTNSKNSEDLSKCESRSYG

>SsGRF3_Sspon.02G0007980-3D

MAAEGEGKNPAGGGGGDNPQHQQAVQAAPVPVPQGEAAQEAGGEGTELEPEGEKADREVEGGGAGEKDDATCRDLVLVEDPEVVAVEDPEEAAATAALQEEMKALVASIPDGAGAAFTAMQLQELEQQSRVYQYMAARVPVPTHLVFPVWKSVTVMGLATLCLDFGKNPEPEPGRCRRTDGKKWRCWRSTIPNEKYCERHMHRGRKRPVQVVVEDDEPDSASGSKSTPGKVTEGAKKADDKSPSSKKLAVAAPAAVQST

>SsGRF4_Sspon.04G0023310-2C

MAMPYASLSPAGADHRSSTATASLLPFCRSTPLSAGGGGLGEDAQVSARWPAARPVMPFTPAQYEELEQQALIYKYLVAGVPVPPDLVVPIRRGLDSLATRFYGHPTLGYATYFGKKLDPEPGRCRRTDGKKWRCSKEAAPDSKYCERHMHRGRNRSRKPVETQLVPQSQPPAATAAVTAALPLAAAAAATNGSSFQNHSLYPAIAGSTGGGGGASNISTPFSSSMGSSQLHMDNAASYAALGGGTAKDLRYNAYGIRSLAEEHNQLISEAIDSSMENQWRLPPSQTSSFPLSSYPQLGALSDLGQSTVSSLSKMERQQPLSFLGNSDFGAMDSAAKQENQTLRPFFDEWPKARDSWPGLSDENTSLACSFPATQLSMSIPMASSDFSVPSSQSPN

>SsGRF5_Sspon.04G0006300-1A

MAEDKETESPQPPAKLPRLSCADTSAGEVTMAASSPLVLGLGLGLGGGGGGSGERDVDSSPATATATPTPKRPSALTFMQQQELEHQVLIYRYFAAGAPVPVHLVLPIWKSVAASSCGPQRFPSRFAIWNECVAVMGLGSLCFDYRSSMEPEPGRCRRTDGKKWRCSRDVVPGHKYCERHVHRGRGRSRKPVEAAAATATAPAAALATSAAAASSLGGGGPVHRGAAPPHPHPHGLGLSSPTSVLLAHSAA

>SsGRF6_Sspon.08G0012220-1P

MMMMSGRAGGGATTAGRYPFTASQWQELEHQALIYKCLASGKPIPSYLMPPLRRILDSALATSPSLAFPPQPSLGWGCFGMGFTRKPDEDPEPGRCRRTDGKKWRCSKEAYPDSKYCEKHMHRGKNRSRKPVEMSLATPAPPASAASSATSATATAATTTSSPAPSYHHRPAPAAHDAAPYHALYGGGGSPYSASSARPAGGAGAYHHHAHLSPFHLHLETTHPHPPPSYYLVDQRDYAYGHATKEVVGEHAFFSDGGAAERDRQHAAGQWQFKQLGMDTKPSPTSLFPIAGYGNNGAASPYGVDLGAKEDEEEERRRQQQQHCFVLGADLRLERPSGHDAAPAQKPLRPFFDEWPHEKGNKAGSWMGLDGETQLSMSIPMAANDLPVTSRYRNGAH

>SsGRF7_Sspon.06G0015340-1A

MEGGGRDVFLGPPPPPPSCPFHGSATAARSGGAQMLSFSSSNGAAGLGLCSGASKMQSVLSRVRGPFTPTQWMELEHQALIYKHFAVNAPVPSSLLLPIKRSLNPWSSLGSSSLGWAPFRSGSGDAEPGRCRRTDGKKWRCSRDAVGDQKYCERHIKRGCHRSRKHVEGRKATPTIADPTMAVSGGLLLHSHAVAWLQQAKSSAANVTDPFSLGSNRNLLDKQNIGDQFSVSTSMDSFDVSASHSSPNHGKVAFSPVEMQHEHDQLYLVHGAGSSAEHVNKSQDGQLLVSRETIDDGPLGEVFKGKSCQSESADILTDQWTST

>SsGRF8_Sspon.05G0023450-1B

MAMPFASLSPAADHRPSSLLPFCRAAPLSAVGEDAAQHTMSSRWAARPALFTAAQYEELEHQALIYKYLVAGVPVPPDLLAPPTPGLPLPPTRPKDETRFIFVGYGTYFGKKVDPEPGRCRRTDGKKWRCSKEAAPDSKYCERHMHRGRNRSRKPVEAQLVPPPHAPQQQQQQQQQPAPAAGFQNHALYPSVLAGNGGGGVVGGGGGTFGMGPTSQLHMDSAAAYATAAGGGSKDLRYSAYGMKSLSDDHSHLLPGGMDTSMDNSWRLLPSQTTTFQATSYPLFGTLSGLDESTIASLPKTQREPLSFFGSDFVTAKQENQTLRPFFDEWPKSRDSWPELAEDNSLGFSATQLSISIPMATSDFSNTSSRSPNGIPSR

>SsGRF9_Sspon.08G0017060-1A

MLSSASSAGAAMGMGGGYPHQPPPLPLPQRGAAAAVFTAAQWAELEQQALIYKYLMAGVPVPPDLLIPVRPSPHSAAFSFASPAASPFYHHHHHPSLSYYAYYGKKLDPEPWRCRRTDGKKWRCSKEAHPDSKYCERHMHRGRNRSRKPVESKTASPAHSSQPQLSTVTTTTREAAAPLESLATAGGKTHGLSLGGGAGSSHLNVDASNAHYRYGSKYPLGAKSDAGELSFFSGASGNSRGFTIDSPSDNSWHSLPSNVPPFTLSKGRDSGLLPGAYSYSHIEPPQELSQVTIASLSQEQERQPFSSGGAGAGGGLLGNVKQENQPLRPFFDEWPGTRDSWSEMDDARSNRTSFSTTQLSISIPMPRCEFPTDPVIENEARAATRWCCRMTLTSIALAASEPSPGD

>SsGRF10_Sspon.08G0012220-1A

MMLSGHGGGRRLFTASQWQELEHQALIFKYMASGAPVPHDLVLPLRLATGVDTAPSLAFPPQHSPSLAYWGCYGAGAPFGRKAEDPEPGRCRRTDGKKWRCSREAHGESKYCEKHIHRGKSRSRKPVEVTSSATSPAAAAYRPSALSISPPRAADAPPPSLGHSQQHLRHGASSAAARAPAQATAGGALQLHLDASLHAASPPPSYHRYAHSHAHYTTPTPSLFPGGGGGYGYDYGQSKELREAELRRRHFHTLGADLSLDKPLPLAATGSDAAAAEKPLRRFFDEWPRESGDTRPSWAGAEDATQLSISIPAASPSDLAASAAARYHNGEPASERLHFYWFLAYNQFADK

>AtGRF1_AT2G22840

MDLGVRVSGHETVSSPGQTELGSGFSNKQERSGFDGEDCWRSSKLSRTSTDGFSSSPASAKTLSFHQGIPLLRSTTINDPRKGQEHMLSFSSASGKSDVSPYLQYCRNSGYGLGGMMNTSNMHGNLLTGVKGPFSLTQWAELEQQALIYKYITANVPVPSSLLLSLKKSFFPYGSLPPNSFGWGSFHLGFSGGNMDPEPGRCRRTDGKKWRCSRDAVPDQKYCERHINRGRHRSRKPVEGQNGHNTNAAAAASAAAASTAAAVSKAAAGTSAVAMRGSDNNNSLAAAVGTQHHTNNQSTDSLANRVQNSRGASVFPATMNLQSKETHPKQSNNPFEFGLISSDSLLNPSHKQASYATSSKGFGSYLDFGNQAKHAGNHNNVDSWPEELKSDWTQLSMSIPMAPSSPVQDKLALSPLRLSREFDPAIHMGLGVNTEFLDPGKKTNNWIPISWGNNNSMGGPLGEVLNSTTNSPKFGSSPTGVLQKSTFGSLSNSSSASSTIIGDNNNKNGDGKDPLGPTTLMNTSATAPSL

>AtGRF2_AT4G37740

MDIGVHVLGSVTSNENESLGLKELIGTKQDRSGFIGEDCLQRSLKLARTTTRAEEEENLSSSVAAAYCKTMSFHQGIPLMRSASPLSSDSRRQEQMLSFSDKPDALDFSKYVGLDNSSNNKNSLSPFLHQIPPPSYFRSSGGYGSGGMMMNMSMQGNFTGVKGPFTLTQWAELEQQALIYKYITANVPVPSSLLISIKKSFYPYGSLPPSSFGWGTFHLGFAGGNMDPEPGRCRRTDGKKWRCSRDAVPDQKYCERHINRGRHRSRKPVEVQSGQNQTAAAASKAVTTPQQPVVAGNTNRSNARASSNRSLAIGSQYINPSTESLPNNRGVSIYPSTVNLQPKESPVIHQKHRNNNNPFEFGHISSDSLLNPNTAKTYGSSFLDFSSNQEKHSGNHNHNSWPEELTSDWTQLSMSIPIASSSPSSTHNNNNAQEKTTLSPLRLSRELDLSIQTDETTIEPTVKKVNTWIPISWGNSLGGPLGEVLNSTTNSPTFGSSPTGVLQKSTFCSLSNNSSVSSPIAENNRHNGDYFHYTT

>AtGRF3_AT2G36400

MDLQLKQWRSQQQQQHQTESEEQPSAAKIPKHVFDQIHSHTATSTALPLFTPEPTSSKLSSLSPDSSSRFPKMGSFFSWAQWQELELQALIYRYMLAGAAVPQELLLPIKKSLLHLSPSYFLHHPLQHLPHYQPAWYLGRAAMDPEPGRCRRTDGKKWRCSRDVFAGHKYCERHMHRGRNRSRKPVETPTTVNATATSMASSVAAAATTTTATTTSTFAFGGGGGSEEVVGQGGSFFFSGSSNSSSELLHLSQSCSEMKQESNNMNNKRPYESHIGFSNNRSDGGHILRPFFDDWPRSSLQEADNSSSPMSSATCLSISMPGNSSSDVSLKLSTGNEEGARSNNNGRDQQNMSWWSGGGSNHHHHNMGGPLAEALRSSSSSSPTSVLHQLGVSTQAFH

>AtGRF4_AT3G52910

MDLQLKQWRSQQQNESEEQGSAATKISNFFFDQIQSQTATSAAAAPLPLFVPEPTSSSSFSCFSPDSSNSSSSSRFLKMGNFFSWAQWQELELQALIYRYMLAGASVPQELLLPIKKSLLHQSPMHFLHHPLQHSFPHHQPSWYWGRGAMDPEPGRCKRTDGKKWRCSRDVVAGHKYCDRHIHRGRNRSRKPVETATTTITTTATTTASSFVLGEELGHGPNNNHFFSSGSSQPLHLSHQQSCSSEMKQESNNNKRPYEANSGFSNGRSDDGHILRHFFDDWPRSSDSTSSPMSSSTCHLSISMPGNNTSSDVSLKLSTGNEEEEENMRNNNNEREQMNWWSNGGNHHNNMGGPLAEALRSASSTSSVLHQMGISTQVFH

>AtGRF5_AT3G13960

MMSLSGSSGRTIGRPPFTPTQWEELEHQALIYKYMVSGVPVPPELIFSIRRSLDTSLVSRLLPHQSLGWGCYQMGFGRKPDPEPGRCRRTDGKKWRCSREAYPDSKYCEKHMHRGRNRARKSLDQNQTTTTPLTSPSLSFTNNNNPSPTLSSSSSSNSSSTTYSASSSSMDAYSNSNRFGLGGSSSNTRGYFNSHSLDYPYPSTSPKQQQQTLHHASALSLHQNTNSTSQFNVLASATDHKDFRYFQGIGERVGGVGERTFFPEASRSFQDSPYHHHQQPLATVMNDPYHHCSTDHNKIDHHHTYSSSSSSQHLHHDHDHRQQQCFVLGADMFNKPTRSVLANSSRQDQNQEEDEKDSSESSKKSLHHFFGEDWAQNKNSSDSWLDLSSHSRLDTGS

>AtGRF6_AT2G06200

MATRIPFTESQWEELENQALVFKYLAANMPVPPHLLFLIKRPFLFSSSSSSSSSSSFFSPTLSPHFGWNVYEMGMGRKIDAEPGRCRRTDGKKWRCSKEAYPDSKYCERHMHRGKNRSSSRKPPPTQFTPNLFLDSSSRRRRSGYMDDFFSIEPSGSIKSCSGSAMEDNDDGSCRGINNEEKQPDRHCFILGTDLRTRERPLMLEEKLKQRDHDNEEEQGSKRFYRFLDEWPSSKSSVSTSLFI

>AtGRF7_AT5G53660

MDFLKVSDKTTIPYRSDSLFSLNQQQYKESSFGFRDMEIHPHPTPYAGNGLLGCYYYYPFTNAQLKELERQAMIYKYMIASIPVPFDLLVSSPSSASPCNNKNIAGDLEPGRCRRTDGKKWRCAKEVVSNHKYCEKHLHRGRPRSRKHVEPPYSRPNNNGGSVKNRDLKKLPQKLSSSSIKDKTLEPMEVSSSISNYRDSRGSEKFTVLATTEQENKYLNFIDVWSDGVRSSEKQSTTSTPVSSSNGNLSLYSLDLSMGGNNLMGQDEMGLIQMGLGVIGSGSEDHHGYGPYGVTSSLEEMSSWLAPMSTTPGGPLAEILRPSTNLAISGDIESYSLMETPTPSSSPSRVMKKMTSSVSDESSQV

>AtGRF8_AT4G24150

MGTRAERKEDFVGGFGFGVVENSHKDVMVLPHHHYYPSYSSPSSSSLCYCSAGVSDPMFSVSSNQAYTSSHSGMFTPAGSGSAAVTVADPFFSLSSSGEMRRSMNEDAGAAFSEAQWHELERQRNIYKYMMASVPVPPELLTPFPKNHQSNTNPDVTVAVATGGSLQLGIASSASNNTADLEPWRCKRTDGKKWRCSRNVIPDQKYCERHTHKSRPRSRKHVESSHQSSHHNDIRTAKNDTSQLVRTYPQFYGQPISQIPVLSTLPSASSPYDHHRGLRWFTKEDDAIGTLNPETQEAVQLKVGSSRELKRGFDYDLNFRQKEPIVDQSFGALQGLLSLNQTPQHNQETRQFVVEGKQDEAMGSSLTLSMAGGGMEETEGTNQHQWVSHEGPSWLYSTTPGGPLAEALCLGVSNNPSSSTTTSSCSRSSS

>AtGRF9_AT2G45480

MQSPKMEQEEVEEERMRNKWPWMKAAQLMEFRMQALVYRYIEAGLRVPHHLVVPIWNSLALSSSSNYNYHSSSLLSNKGVTHIDTLETEPTRCRRTDGKKWRCSNTVLLFEKYCERHMHRGRKRSRKLVESSSEVASSSTKYDNTYGLDRYNESQSHLHGTISGSSNAQVVTIASLPSARSCENVIRPSLVISEFTNKSVSHGRKNMEMSYDDFINEKEASMCVGVVPLQGDESKPSVQKFFPEVSDKCLEAAKFSSNRKNDIIARSREWKNMNVNGGLFHGIHFSPDTVLQERGCFRLQGVETDNEPGRCRRTDGKKWRCSKDVLSGQKYCDKHMHRGMKKKHPVDTTNSHENAGFSPLTVETAVRSVVPCKDGDDQKHSVSVMGITLPRVSDEKSTSSCSTDTTITDTALRGEDDDEEYLSLFSPGV

>OsGRF1_LOC_Os02g53690.1

MMMMSGRPSGGAGGGRYPFTASQWQELEHQALIYKYMASGTPIPSDLILPLRRSFLLDSALATSPSLAFPPQPSLGWGCFGMGFGRKAEDPEPGRCRRTDGKKWRCSKEAYPDSKYCEKHMHRGKNRSRKPVEMSLATPPPPSSSATSAASNTSAGVAPTTTTTSSPAPSYSRPAPHDAAPYQALYGGPYAAATARTPAAAAYHAQVSPFHLQLDTTHPHPPPSYYSMDHKEYAYGHATKEVHGEHAFFSDGTEREHHHAAAGHGQWQFKQLGMEPKQSTTPLFPGAGYGHTAASPYAIDLSKEDDDEKERRQQQQQQQQQHCFLLGADLRLEKPAGHDHAAAAQKPLRHFFDEWPHEKNSKGSWMGLEGETQLSMSIPMAANDLPITTTSRYHNDD

>OsGRF2_LOC_Os06g10310.1

MMAGGGSGRCLFTATQWQELEHQALIYKYMAAGAPVPPDLLLHLRHRAAAAAAADVDTVPSLAFPPHHLGWGCYGAAAAQYGRRVEDPEPGRCRRTDGKKWRCSREAYGESKYCEKHMHRGKNRSRKPVEMPPPAAAAVYRPSALSISPPPHDADAPSYGAGAGAPLQLHLDSFHASTSPPPSYHRYAHTSSAPLFPSSAAGYGGGWSLSKEHCLTLGGAAADLSLDKPADHHHDATSATTEKPLRRFFDEWPRSDDGRTPWDGTQLSISIPTAAAASPDLAIAGAASRYHSNGDHLRTSE

>OsGRF3_LOC_Os04g51190.1

MAMPFASLSPAADHRPSFIFPFCRSSPLSAVGEEAQQHMMGARWAAAVARPPPFTAAQYEELEQQALIYKYLVAGVPVPADLLLPIRRGLDSLASRFYHHPVLGYGSYFGKKLDPEPGRCRRTDGKKWRCSKEAAPDSKYCERHMHRGRNRSRKPVEAQLVAPHSQPPATAPAAAVTSTAFQNHSLYPAIANGGGANGGGGGGGGGGSAPGSFALGSNTQLHMDNAASYSTVAAGAGNKDFRYSAYGVRPLADEHSPLITGAMDTSIDNSWCLLPSQTSTFSVSSYPMLGNLSELDQNTICSLPKVEREPLSFFGSDYVTVDSGKQENQTLRPFFDEWPKARDSWPDLADDNSLATFSATQLSISIPMATSDFSTTSSRSHNGIYSR

>OsGRF4_LOC_Os02g47280.1

MAMPYASLSPAVADHRSSPAAATASLLPFCRSTPLSAGGGGVAMGEDAPMTARWPPAAAARLPPFTAAQYEELEQQALIYKYLVAGVPVPPDLVLPIRRGLDSLAARFYNHPALGYGPYFGKKLDPEPGRCRRTDGKKWRCSKEAAPDSKYCERHMHRGRNRSRKPVETQLVAQSQPPSSVVGSAAAPLAAASNGSSFQNHSLYPAIAGSNGGGGGRNMPSSFGSALGSQLHMDNAAPYAAVGGGTGKDLRYTAYGTRSLADEQSQLITEAINTSIENPWRLLPSQNSPFPLSSYSQLGALSDLGQNTPSSLSKVQRQPLSFFGNDYAAVDSVKQENQTLRPFFDEWPKGRDSWSDLADENANLSSFSGTQLSISIPMASSDFSAASSRSTNGD

>OsGRF5_LOC_Os06g02560.1

MLSSSPSAAAPGIGGYQPQRGAAVFTAAQWAELEQQALIYKYLVAGVPVPGDLLLPIRPHSSAAATYSFANPAAAPFYHHHHHPSLSYYAYYGKKLDPEPWRCRRTDGKKWRCSKEAHPDSKYCERHMHRGRNRSRKPVESKTAAPAPQSQPQLSNVTTATHDTDAPLPSLTVGAKTHGLSLGGAGSSQFHVDAPSYGSKYSLGAKADVGELSFFSGASGNTRGFTIDSPTDSSWHSLPSSVPPYPMSKPRDSGLLPGAYSYSHLEPSQELGQVTIASLSQEQERRSFGGGAGGMLGNVKHENQPLRPFFDEWPGRRDSWSEMDEERSNQTSFSTTQLSISIPMPRCGSPIGPRLP

>OsGRF6_LOC_Os03g51970.1

MQGAMARVRGPFTPSQWIELEHQALIYKYLAANSPVPHSLLIPIRRSLTSPYSPAYFGSSTLGWGSFQLGYSGSADPEPGRCRRTDGKKWRCSRDAVADQKYCERHMNRGRHRSRKHVEGQPGHAAKAMPAAVAAAAASATQPSAPAAHSGGAVAGLAINHQHQQMKNYAANTANPCSLQYSRDLANKHNESEQVQDSDSLSMLTSISTRNTGSLFPFSKQHNPFEVSNSRPDFGLVSPDSLMSSPHSSLENVNLLTSQSLNEQQSSVSLQHFVDWPRTPAQGALAWPDAEDMQAQRSQLSISAPMASSDLSSASTSPIHEKLMLSPLKLSREYSPIGLGFAANRDEVNQGEANWMPMFRDSLMGGPLGEVLTKNNNMEARNCLSESLNLLNDGWDSSSGFDSSPVGVLQKTTFGSVSSSTGSSPRLENHSVYDGNSNLRDDLGSVVVNHPSIRLV

>OsGRF7_LOC_Os12g29980.1

MAMATPTTNGSFLLGSGGYPGAQILSFSSSGHSGNGLDCGSSDVARMQGVLARVRGPFTPTQWMELEHQALIYKHIVANAPVPAGLLLPIRRSLHPPVFPHFSSGGILGSSSLGWGSFQLGYSGSADSEPGRCRRTDGKKWRCSRDAVVDQKYCERHINRGRHRSRKHVEGQSSHAAKATVPAIAQPPIGASNGKLSGSHGVSNELTKTLATNRMMLDKANLIERSQDYTNQQHNILQNNTKGDNWSEEMSSQADYAVIPAGSLMNTPQSANLNPIPQQQRCKQSLFGKGIQHDDIQLSISIPVDNSDLPTNYNKAQMDHVVGGSSNGGNNTRASWIPGSWEASIGGPLGEFFTNTSSASDDKGKSRHPPSLNLLADGHTTSPQLQSPTGVLQMTSFSSVPSSTVSSPAGSLCNGLLTSGLVNAQTVQTL

>OsGRF8_LOC_Os11g35030.1

MLSSCGGHGHGNPRSLQEEHHGRCGEQQGGGGGGGQEQEQDGFLVREARASPPSPSSSSFLGSTSSSCSGGGGGGQMLSFSSPNGTAGLGLSSGGSMQGVLARVRGPFTPTQWMELEHQALIYKHIAANVSVPSSLLLPIRRSLHPWGWGSFPPGCADVEPRRCRRTDGKKWRCSRDAVGDQKYCERHINRGRHRSRKHVEGRKATLTIAEPSTVIAAGVSSRGHTVARQKQVKGSAATVSDPFSRQSNRKFLEKQNVVDQLSPMDSFDFSSTQSSPNYDNVALSPLKLHHDHDESYIGHGAGSSSEKGSMMYESRLTVSKETLDDGPLGEVFKRKNCQSASTEILTEKWTENPNLHCPSGILQMATKFNSISSGNTVNSGGTAVENLITDNGYLTARMMNPHIVPTLL

>OsGRF9_LOC_Os03g47140.1

MFADFSAAAMELGEVLGLQGLTVPSTKEGDLSLIKRAAAGSFTQAAAASYPSPFLDEQKMLRFAKAAHTLPSGLDFGRENEQRFLLSRTKRPFTPSQWMELEHQALIYKYLNAKAPIPSSLLISISKSFRSSANRMSWRPLYQGFPNADSDPEPGRCRRTDGKKWRCSKEAMADHKYCERHINRNRHRSRKPVENQSRKTVKETPCAGSLPSSVGQGSFKKAKVNEMKPRSISYWTDSLNRTMANKEKGNKAAEENNGPLLNLTNQQPTLSLFSQLKQQNKPEKFNTAGDSESISSNTMLKPWESSNQQNNKSIPFTKMHDRGCLQSVLQNFSLPKDEKMEFQKSKDSNVMTVPSTFYSSPEDPRVSCHAPNMAQMQEDSISSSWEMPQGGPLGEILTNSKNPDDSIMKPEARPYGWLLNLEDHAM

>OsGRF10_LOC_Os02g45570.1

MDEEKEADSPQPPSKLPRLSGADPNAGVVTMAAPPPPVGLGLGLGLGGDSRGERDVEASAAAAHKATALTFMQQQELEHQVLIYRYFAAGAPVPVHLVLPIWKSVASSSFGPHRFPSLAVMGLGNLCFDYRSSMEPDPGRCRRTDGKKWRCSRDVVPGHKYCERHVHRGRGRSRKPVEASAAATPANNGGGGGIVFSPTSVLLAHGTARAT

>OsGRF11_LOC_Os07g28430.1

MAAEGEAKKDSASNPPGGGGGGGGGEEEEDSSLAVGEAAVGVGEAGGGGGGGEKADREEEEGKEDVEEGGVCKDLVLVEDAVPVEDPEEAAATAALQEEMKALVESVPVGAGAAFTAMQLQELEQQSRVYQYMAARVPVPTHLVFPIWKSVTGASSEGAQKYPTLMGLATLCLDFGKNPEPEPGRCRRTDGKKWRCWRNAIANEKYCERHMHRGRKRPVQLVVEDDEPDSTSGSKPASGKATEGGKKTDDKSSSSKKLAVAAPAAVEST

>OsGRF12_LOC_Os04g48510.1

MLAEGRQVYLPPPPPSKLPRLSGTDPTDGVVTMAAPSPLVLGLGLGLGGSGSDSSGSDAEASAATVREARPPSALTFMQRQELEQQVLIYRYFAAGAPVPVHLVLPIWKSIAAASSFGPQSFPSLTGLGSLCFDYRSSMEPEPGRCRRTDGKKWRCSRDVVPGHKYCERHVHRGRGRSRKPMEASAAVAPTYLPVRPALHTVATLATSAPSLSHLGFSSASKVLLAHTTTGTTRAT

>ZmGRF1_GRMZM2G178261

MDLGGSVVMDAGASELGLLGGGSSRLLKHGRGPNAAGGDDDVWGGGRAKQPRTGTAPVVAGDVAAATKAAAAPFLLGSCSPGHGGEQMLSFSSAAAAAAASCASSTAAADGGGAMPLYYGTPASCSGLSSVSLSTSMQGAMARVRAPFTPSQWIELEHQALIYKYLAANSPIPHSLLIPIRRSLAASPYPPSYFGTSTLGWGSFQLGYSGNADLEPGRCRRTDGKKWRCSRDAVADQKYCERHMNRGRHRSRKHVEGQPGHAAKAMSAVAAAAAAAAQPAALAGAGAPAAGLTVNQHQLPAKSYATGATDPCSLQYNRELVNKQNESENMQDSDNLSMLTSMNTGNTGSVFPFSKQNTNPFEVTSSRLEFGLVSSDSLMSSPHSSLENVNLLGSQSMNEHQNPASLQHFVDWPRTPAQGGLSWPEAEDMQAQRSQLSISAPMASSELSSASTSPIHEKLMLSPLKLSREYSPTGLGIAANRDEASQLEATWATMFRDSSMGGPLGEVLAKNGNVEARNYLSAPLNLLTDYWDSSHGMESSPVGVLQKTTAFGSVSSSTGSSPRVENHGAYDGFGNLRDDLGSIVVRHPSIRLV

>ZmGRF2_GRMZM2G018414

MSAEFCAAAAGAVAMELGVGDVMGLQQGIAAATGPSSGDSDLGLLKRAGLAQAATSYPSPFLDQQKMLRFSKAAAAHTSPSGLDFGGGPSEQAFLLSRTKRPFTPSQWMELEHQALIYKYLNAKAPIPSSLLVSISKSFRSSNRVSWRPLYQGYANADSDPEPGRCRRTDGKKWRCSKEAMPDHKYCERHINRNRHRSRKPVENQPRKATKEVTTAAAGSLPCAGPQGSLKKAKVNDSKPGTGSYWTDSLNRTMLSREKANKPTDDESLLLSSTKNSQPTLSLLTQLKQQNKPDKLGPTPENEPNSDTMLKAWGGSHHKSISSTQRHDAESLQSVLQNFSLAQNDRLESEKNRYSDSVLVSSAFYSADGPQTTCLTPNMTQVQQDCISSSWEMPQGGPLGEILTNSKISEDLSKCGSRSYGWLLNLDHAP

>ZmGRF3_GRMZM2G041223

MAMPFASLSPAADHRPSSLLPYCRAAPLSAVGEDAAAQAQQQQQHAMSGRWAARPPALFTAAQYEELEHQALIYKYLVAGVPVPPDLLLPLRRGFVYHQPALGYGPYFGKKVDPEPGRCRRTDGKKWRCSKEAAPDSKYCERHMHRGRNRSRKPVEAQLVPPPHAQQQQQQQAPAPTAGFQSHPMYPSILAGNGGGGGGVGGGAGGGGTFGLGPTSQLHMDSAAAYATAAGGGSKDLRYSAYGVKSLSDEHSQLLSGGGGMDASMDNSWRLLPSQTAATFQATSYPLFGALSGLDESTIASLPKTQREPLSFFGSDFVTPKQENQTLRPFFDEWPKSRDSWPELNEDNSLGSSATQLSISIPMAPSDFNTSSRSPNGIPSR

>ZmGRF4_GRMZM2G004619

MAAEGEAKNPSGGGEGGNPQHQQAVQAAPAEPPTAQGEAVQEAGAQATGQEPEGEKANRDGEGSAGEKDDGACRDLVLVEDPEVLAVEDPEEAAATAALQEEMKALVASVPDGAGAAFTAMQLQELEQQSRVYQYMAARVPVPTHLVFPVWKSVTGASSEGAQKYPTLLGLATLCLDFGKNPEPEPGRCRRTDGKKWRCWRNTIPNEKYCERHMHRGRKRPVQVVEEAEPDSASGSKSAPSKATEGAKKVDDKSPGSKKLAVAAAAAAAVQST

>ZmGRF5_GRMZM2G099862

MEGGRDVFLGAAARAPPPPPSCPFHGSATATRSGGAQMLSFSSNGVAGLGLCSGASKMQGVLSRVRRPFTPTQWMELEHQALIYKHFAVNAPVPSSLLLPIKRSLNPWSSLGSSSLGWAPFRSGSADAEPGRCRRTDGKKWRCSRDAVGDQKYCERHIKRGCHRSRKHVEGRKATPTTADPTMAVSGGSLLHSHAVAWQQQGKSSAANVTDPFSLGSNRNLLDKQNLGDQFSVSTSMDSFDFSSSHSSPNQAKVAFSPVAMQHEHDQLYLVHGAGSSAENVNKSQDGQLLVSRETIDDGPLGEVFKGKSCQSASADILTDHWTSTRDLRPPTGVLQMSSSNTVPAENHTSNSSYLMARMANSQTVPTLH

>ZmGRF6_GRMZM2G124566

MAEDKETDSPQPPAKLPRLSRADTSAGKHARAGSREVTMAASSPLVLGLGLGLGGRGGGGERDADVSPATVTPKRPSALTFMQQQELEHQVLIYRYFAAGAPVPVHLVLPIWKSVAASSFGPQRFPSLMGLGSLCFDYRSSMEPEPGRCRRTDGKKWRCSRDVVPGHKYCERHVHRGRGRSRKPVEAAAAPAAAAGGSSPVLRVAAPQHVLGLSSPTSVLLAHGAARAT

>ZmGRF7_GRMZM2G105335

MMMMSSGRAGGGATAGRYPFTASQWQELEHQALIYKCLASGKPIPSYLMPPLRRILDSALATSPSLAYPPQPSLGWGCFGMGFTRKADEDPEPGRCRRTDGKKWRCSKEAYPDSKYCEKHMHRGKNRSRKPVEMSLATPAPAPAPAAATTATATSSPAPSYHRPAHDATPSPYHALYGGGGGGGGSPYSASARPGATGGGGAYHHAQHVSPFHLHLETTHPHPPPPYNYSADQRDYAYGHAAAKEVGEHAFFSDGAGERVDRQAAAGQWQFRQLGVETKPGPTPLFPVAGYGHGAASPYGVEMGKDDDEQEERRRQHCFVLGADLRLERPSSGHGHGHDHDDAAAAQKPLRPFFDEWPHQKGDKAGSWMGLDGETQLSMSIPMAATDLPVTSRFRNGGHYE

>ZmGRF8_GRMZM5G853392

MSSARVRGPFTPPQWIELEHQALIYKYLSANSPVPHSLLVPIRRSGSLASSPYPPPYFGTSTFGWGSFQLGYSGNADLEPGRCRRTDGKKWRCSRDAVADQKYCERHMNRGRHRSRKHVEDQPGHAAKAMSAVAVAAAAAAATQHAAALAGAGATAAAGLAVNRHRQPGKSYATGATDPCSLQYSRELANKLSNESENMQDSDNVSMLTSMNTGNTAGSVFPFSKQNTALFEVSSSRPEFGLVPSDSLTSSPHSSLKSVKNLLSSRSLSEHQSSASLQHFVDWPRTPPAQGGLSWPEAEDMPRAQRSQLSVSAPMASSELSSASTSPIHDHEKLILSPLKLGREYTPSGVVSAAANRYEVSQLEATWATMFRDSSTGGPLGEVLAKNNSNAEARGCPSAAPPLTDYYCWDSGHGMQSSPVGVLQISVQL

>ZmGRF9_GRMZM2G045977

MAAEDKETDSPQPPAKLPRLSCADTSAGVAASSPLVLGLGLGLGGGGERDADASPATATPKRPSALTFMQQQELEHQVLIYRYFAAGAPVPAHLVLPIWKSVAASSFGPQRFPSLMGLGSLCFDYRSSMEPEPGRCRRTDGKKWRCSRDVVPGHKYCERHVHRGRGRSRKPVEAAAPAPSAAAATSLGGGPVHHTGGAPPPHGLGLSPTSVLLAHSAARAT

>ZmGRF10_GRMZM2G034876

MAMPYASLSPAGAADHRSSTATASLVPFCRSTPLSAGGGLGEEDAQASARWPAARPVVPFTPAQYQELEQQALIYKYLVAGVPVPPDLVVPIRRGLDSLATRFYGQPTLGYGPYLGRKLDPEPGRCRRTDGKKWRCSKEAAPDSKYCERHMHRGRNRSRKPVETQLAPQSQPPAAAAVSAAPPLAAAAAATTNGSGFQNHSLYPAIAGSTGGGGGVGGSGNISSPFSSSMGGSSQLHMDSAASYSYAALGGGTAKDLRYNAYGIRSLADEHNQLIAEAIDSSIESQWRLPSSSFPLSSYPHLGALGDLGGQNSTVSSLPKMEKQQPPSSFLGNDTGAGMAMGSASAKQEGQTLRHFFDEWPKARDSWPGLSDETASLASFPPATQLSMSIPMASSDFSVASSQSPNGESRTFLLATDRR

>ZmGRF11_GRMZM2G098594

MLSSASSAAGAAMGMGGGGYAHQPPPQRTVFTAAQWAELEQQALIYKYLMAGVPVPPDLLLPVRPGPAAAFSFAGPAAASPFYHQHHPSLSYYAYYGKKLDPEPWRCRRTDGKKWRCSKEAHPDSKYCERHMHRGRNRSRKPVESKTASSSSPAHPSPPQLSTVTTTAPLEPLAAAGGKVHGLSLGGGAAGSSHLGVDASNAHYRYGSNRYPLGAKPDGGELSFFSGASSGNNSRGGFTIDSPSDNNSWHSALASSVPPFTLSTKSGDSGLLPGAYASYSQSHSHMEPPRELGQVTIASLAQEQERQQPFSGGMLGNVKQENQNQPLRPFFDEWPGTRADSWPPEMDGAPRAGRTSFSSSTTQLSISIPMPRCELHLRNQNS

>ZmGRF12_GRMZM5G850129

MMLSGHGGGRRLFTASQWQELEHQALIFKYMASGAPVPHDLVLPLRLATGVDTAPSLAFPPQPSPSRTTCALLSSRPDLPRPRLMFHSDHRQPSAHHIIADDFVRVTVHAVAYWGCYGAGAPFGRKAAEDTEPGRCRRTDGKKWRCSREAHGDSKYCEKHIHRGKSRSRKPVEVTSSPAAGPAAAYRPSAISTISPPRAADAPPPSLAYPQQHLLHGASSAAGAAARVPAGALQLHLDASLHAAAAAASPSPPPSYHRYAHYTPPASSLFPGGGYGYDYDYGQSKELRRRHFHALGADLSLDKPLPEPDTGSDEKQPLRRFFDEWPRESGDMAADDATQLSISIPAASPSDLAATSASAAAARFHNGEAASQRLHFHWFLAYDRIADWCCRFIIV

>ZmGRF13_GRMZM2G096709

MTAEGEAKNPSAGGGGDNPQHQQAAPAPAPAQGEVAQEAAVQGTGQEQERDKADREVQGGAGEKDDGACRDLVLVEDPEVLAVEDPEEAAATAALQEEMKALVASIPDGAGAAFTAMQLQELEQQSRVYQYMAARVPVPTHLVFPVWKSVTGASSEGAQKYPTLMGLATLCLDFGKNPEPEPGRCRRTDGKKWRCWRNTIPNEKYCERHMHRGRKRPVQVFLEDDEPDSASGSKPAAPGKATEGAKKADDKSPSSKKLAVAAPAAVQST

>ZmGRF14_GRMZM2G067743

MMLSGHGGGRRLFTASQWQELEHQALIFKYMASGAPVPHDLVLPLRLATGVDTAPSLAFPPQPSPSLAYWGCYGAGAPFGRKAEDPEPGRCRRTDGKKWRCSREAHGDSKYCEKHIHRGKSRSRKPVEVTSPAAYRPSAFSISPPRAADAPPPPPGLGHPQQQHLRHGALSPAGRAHAAGALQLHLDSSLHAASPPPSYHRYAHSHAHYTPPPPPSLYDYGQSKELREAAELRRRHFHALGADLSLDKPLADAGAAEKPLRRFFDEWPRERGDTRPSWAGAEDATQLSISIPAASPSSDHAASAAARCHNDGSDRCIS

>ZmGRF15_GRMZM2G129147

MGMAMPFASPSPAADHRPSSLLPFCRAAPLSAAGEDAAQQHAMSGRWAARPALFTAAQYEELEHQALIYKYLVAGVPVPPDLLLPLRRGFVFHQPPALGYGPYFGKKVDPEPGRCRRTDGKKWRCSKEAAPDSKYCERHMHRGRNRSRKPVEAQLAPPPHAQPQQQQQAPAPAAGFQNHSLYPSILTGNGGGGVGAGAGGGTFGLGPTSQLHMDSAAAYATAAGGGSKYLRYSAYGVKSLSDEHSTLLSGGMDPSMMDNSWRLLPSQTNTFQATSYPVFGTLSGLDESTIASLPKTQREPLSFFGSDFVTAAKQENQTLRPFFDEWPKSRDSWPELGEDSSLGFSATQLSISIPMATSDFSNTSSRSPGGIPSR

>SbGRF1_Sobic.001G104500.1

MDLMGGVVMDAGGGGAAELGLLGGGGSSSRLLKHGRGNAAAGGEDHGWGGGVGGGRAKQARTATASVVAGDVVEAAKAAAAPFLLGSCSPGHGGEQMLSFSSATAAASSCASTAAVAAAVAADGGAMPLYYGTPASCSGLSSVSLSTSMQGAMARVRGPFTPSQWIELEHQALIYKYLAANSPIPHSLLIPIRRSLASSPYPPSYFGTSTLGWGSFQLGYSGNADLEPGRCRRTDGKKWRCSRDAVADQKYCERHMNRGRHRSRKHVEGQPGHAAKAMSAVVAAAATQPAALAGAGATAAGLTVNQHQQPAKSYATGAADPCSLQYNRELVNKQNESENMQDSDNLSMLTSMTTGNTGSVFPFSKQNNPFEVTSSRPEFGLVSSDSLMSSPHSSLENVNLLSSQSLNEHQSSATLQHFVDWPRTPAQGGLSWPEAEDMQAQRSQLSISAPMASSELSSASTSPIHEKLMLSPLKLSREYSPTGLSIAANRDEVSQLEATWATMFRDSSMGGPLGEVLNKNGNVEAKNCLSAPLNLLTDYWDSSHGMESSPVGVLQKTTFGSVSSSTGSSPRIENHGAYDGISNLRDDLGSIVVSHPSIRFV

>SbGRF2_Sobic.001G139800.1

MSAEFCAAAGGGGAAVAMEFGVVGDVMGLQQGIAVTAPSPRDSSDLGLLKRAALTQAAAAAAAPYPSPFLDEQKMLRFSKAAHTLPSGLDFGGPSEQAFLLSRTKRPFTPSQWMELEHQALIYKYLNAKAPIPSSLLISISKSFRSSNRVSWRPLYQGYTNADSDPEPGRCRRTDGKKWRCSKEAMADHKYCERHINRNRHRSRKPVENQPKKTTKEVPAAAGSLPCAGPQGSLKKAKVNDSKPGTVSYWTDNLNRTMLNREKANKLTEDNSLLLNSTNSQPTLSLLSQLKQQNKPDKLGPTLENESNSDTILKAWGGNQPSHKGISSAQRHDADSLQSVLQNFSLAQNEKMESEKNKYSDSMLVSSTFYSADGPRSTCLTPNMTQVQQDCISSSWEMPQGGPLGEILSNNKNSEDLSKCESRSYGWLLNLDQAP

>SbGRF3_Sobic.002G297800.1

MAAEGEGKNPAGGGGGDNPQHQQAVQAAPAPAAQGEAAQEAGGQGTGLEPEGEKADREGEGGGAGEKDDAACRDLVLVEDPEVVAVEDPEEAAATAALQEEMKALVASIPDGAGAAFTAMQLQELEQQSRVYQYMAARVPVPTHLVFPVWKSVTGASSEGAQKYPTLMGLATLCLDFGKNPEPEPGRCRRTDGKKWRCWRSTIPNEKYCERHMHRGRKRPVQVVVEDDEPDSASGSKSTPGKATDGAKKADDKSPSSKKLAVAAPAAVQST

>SbGRF4_Sobic.004G269900.1

MAMPYASLSPAGADHRSSTATAASLLPFCRSTPLSAGGGGGLGEDAQLSSRWPAARPVVPFTPAQYEELEQQALIYKYLVAGVPVPPDLVVPIRRGLDSLATRFYGHPTLGGYGTYYLGKKLDPEPGRCRRTDGKKWRCSKEAAPDSKYCERHMHRGRNRSRKPVETQLVPQSQPPATAAAVSAAPPLALAAATTTTNGSCFQNHSLYPAIAGSTGGGGGASNISTPFSSSMGSSQLHMDNAASYAALGGGTAKDLRYNAYGIRSLAEEHNQLIAEAIDSSMENQWRLPPSQTSSFPLSSYPQLGALSNLGQSTVTSLSKMERQQPLSFLGNSEFGAMESAAKQQENQTLRPFFDEWPKARDSWPGLSDDNAASLAPSFPATQLSMSIPMASSDFSVASSQSPNDD

>SbGRF5_Sobic.004G282601.1

MAGWPLPVPPQRHSFYSDDHCAPFAFEERTRRPAVTERATRRREPSRINISTASHFPLTPLLSLLPSPLLSSRLVSSREAARALPPLPSPSLHKSWNPNQTAAGSAAQQHRPVFSEMAEDKETESPQPPAKQPRLSCADTSAGEVTMAASSPLVLGLGLGLGASGVGERDADASPATATATATATPKRPSALTFMQQQELEHQVLIYRYFAAGAPVPVHLVLPIWKSVAASSFGPQRFPSRKHRQKLQPPLGVPFLASWMFRV

>SbGRF6_Sobic.004G317000.1

MMMMSGRAGGGATAGRYPFTASQWQELEHQALIYKCLASGKPIPSYLMPPLRRILDSALATSPSLAFPPQPSLGWGCFGMGFSRKPDEDPEPGRCRRTDGKKWRCSKEAYPDSKYCEKHMHRGKNRSRKPVEMSLATPAPASAVSSATSATAAAAAATTTTSSPAPSYRPAPTSHDASPYHALYGGGSPYSASARPAGGPGPYHHPAQVSPFHLHLETTHPHPPPSYYSVDQRDYAYGHATKEVVGEHAFFSDGAAERDRQHAAGQWQFKQLGMDTKPSPTSLFPVAGYGNGAGASPYGVDLGAKEDDEEERRRQQQQHCFVLGADLRLERPSSGHDAATAQKPLRPFFDEWPHEKGNKGGSWMGLDGETQLSMSIPMAASDLPVTSRYRNDE

>SbGRF7_Sobic.005G150900.1

MERGGRDVFLGPTPPPPPSCPFHGSATAARSGGAQMLSFSSSSPSNGAVGLGLCSGASKMQSVLSRVRGPFTPTQWMELEHQALIYKHFAVNAPVPSSLLLPIKRSLNPWSSLGSSSLGWAPFRSGSGDAEPGRCRRTDGKKWRCSRDAVGDQKYCERHIKRGCHRSRKHVEGRKATPTIADPTMVVSGGSLLYSHAVTWQQQAKSSAANVADPFSLGSNRNLLDKQNIGDQFSVSTSMDSFDFSASHSSPNHDKVAFSPVEMQHEHDQLYLVHGAGSSAEHVNKSQDGQLLVSRETIDDGPLGEVFKGKSCQSASADILTDQWTSTCELHSPTGILQMSSSNRVTVENHTSNNSYLMARMVNSHTVPTLH

>SbGRF8_Sobic.006G203400.1

MAMPFASLSPAADHRPSSLLPFCRAAPLSAVGEDAAQHHQQQQQQHTMSGRWAARPALFTAAQYEELEHQALIYKYLVAGVPVPPDLLVPLRRGFVYHQPALGYGTYFGKKVDPEPGRCRRTDGKKWRCSKEAAPDSKYCERHMHRGRNRSRKPVEAPLVPPPHAPQQQQQQQQPPPAPVAGFQNHSLYPSVLAGNGGVGVGGGGGGTFGMGPTSQLHMDSAAAYATAAGGGSKDLRYSAYGAKSLSDDHSQLLPGGMDPSMDNSWRLLPSQTTTFQATSYPVFGTLSGLDESTIASLPKTQREPLSFFGSDYVTAKQENQTLRPFFDEWPKSRESWPELAEDNHLGFSATQLSISIPMATSDFSNTSSRSPNGIPSR

>SbGRF9_Sobic.010G013500.2

MAGVPVPPDLLLPVRPGPHSAAAAFSFAGPAASPFYHHHHHPSLSYYAYYGKKLDPEPWRCRRTDGKKWRCSKEAHPDSKYCERHMHRGRNRSRKPVESKTASPAHPSQPQLSTVTTTTREAAAAAPLESLAAAGGKAHGLSLGGGAGSSHLNVDASNAHYRYGSKYPIGAKSDASELSFFSGASGNSRGFTIDSPSDNSWHSLPSNVPPFTLSKGRDSGLLPGAYSYSHIEPPQELGQVTIASLSQEQERQPFSSGGAGAGAGGLLGNVKQENQPLRPFFDEWPGTRDSWSEMDDARSNRTSFSTTQLSISIPMPRCD

>SbGRF10_Sobic.010G077200.1

MMLSGHGGGRRLFTASQWQELEHQALIFKYMASGAPVPHDLVLPLRLATGVDTAPSLAFPPQPSPSLAYWGCYGAGAPFGRKAEDPEPGRCRRTDGKKWRCSREAHGESKYCEKHIHRGKSRSRKPVEVTSPAAAAYRPSALSISPPRAADAPPPSLAHPHQHLRHGAASAAAAARGAPGQATAGALQLHLDASLHAASPPPSYHRYAHSHAHYTPPPSLFPGGGGGYDYGQSKELREAELRRRHFHALGADLSLDKPLAATAAGSAAAEKPLRRFFDEWPRESGDTRPSSWAGAEDATQLSISIPAASPSDLAASAAARYHNGEPSGERKTPLLLVP
