# Supplementary material for: Genome-wide characterization and expression analysis of the growth-regulating factor family in Saccharum
Source: BMC Plant Biol. 2022 Nov 2;22:510. doi: 10.1186/s12870-022-03891-4 (PMC9628180; doi:10.1186/s12870-022-03891-4)
Supplement: Supplementary file 6 — Supplementary Material 6 [file 12870_2022_3891_MOESM6_ESM.docx]

**Supplementary Table 5:** The primers for RT-qPCR verification of two *GRF* genes in *Saccharum* hybrid YT55 and YT00-236.

| Gene name | Forward primer (5’-3’) | Reverse primer (5’-3’) |
| --- | --- | --- |
| *GRF1* | TGCAACACTTTGTGGACTGG | TGAGCTTGCATGTCTTCTGC |
| *GRF3* | TGAAAGCACTTGTGGCATCG | TCTTCCATACGGGGAAAACGAG |
